# Updated evidence of the Naoshuantong capsule against ischemic stroke: a systematic review and meta-analysis of randomized controlled trials

**DOI:** 10.3389/fphar.2024.1434764

**Published:** 2024-09-26

**Authors:** Cuilin Que, Yupeng Wei, Guanxiang Yin, Congren Zhou, Zhenhong Liu, Xinxing Lai, Mingzhen Qin, Xuejiao Xiong, Xiangyi Zheng, Xinglu Dong, Ying Gao

**Affiliations:** ^1^ Department of Neurology, Dongzhimen Hospital, Beijing University of Chinese Medicine, Beijing, China; ^2^ Institute for Brain Disorders, Beijing University of Chinese Medicine, Beijing, China; ^3^ Beijing University of Chinese Medicine, Beijing, China; ^4^ Chinese Medicine Key Research Room of Brain Disorders Syndrome and Treatment of the National Administration of Traditional Chinese Medicine, Beijing, China

**Keywords:** ischemic stroke, Naoshuantong capsule, meta-analysis, systematic review, Chinese patent medicine

## Abstract

**Background:**

Stroke is a serious health issue that can result in death or disability, leading to a significant economic strain on society and families. A growing number of studies have shown that the Naoshuantong capsule (NSTC) is beneficial as a treatment for ischemic stroke (IS) in recent years. Our study aims to provide an update on the safety and efficacy of the NSTC in IS patients.

**Methods:**

We thoroughly searched eight databases to identify suitable randomized controlled trials (RCTs) assessing the effectiveness of the NSTC in the treatment of IS. The National Institute of Health Stroke Scale (NIHSS) for an acute period and modified Rankin Scale (mRS) at 3 months for a non-acute period were considered the primary outcome, and secondary outcomes included the NIHSS for a non-acute period, mRS, Barthel Index (BI), modified Barthel Index (MBI), Stroke-specific Quality of life (SS-QOL), and the recurrence rate of cerebrovascular events. Subsequently, its quality was assessed using the Cochrane risk assessment scale. Statistical analysis was conducted using RevMan 5.3 and Stata 14.0.

**Results:**

A total of 27 RCTs were included, which involved 3,139 patients. The results showed that the NSTC improved neurological function not only in the acute period (MD = −2.53; 95% CI: −2.91, −2.15; *p* < 0.00001) but also in the non-acute period (MD = −3.70; 95% CI: −5.82, −1.58; *p* = 0.0006) and improved the long-term functional outcomes with lower mRS scores (MD = −0.68; 95% CI: −1.09, −0.26; *p* = 0.001). At the same time, the NSTC decreased the risk of cerebrovascular disease recurrence (RR = 0.43; 95% CI: 0.27, 0.70; *p* = 0.0006) and increased the quality of life in the acute period (MD = 23.88; 95% CI: 16.63, 31.13; *p* < 0.00001). Significant disparities in the incidence of adverse events between the NSTC and control groups were not observed. The certainty of evidence was estimated as moderate to very low.

**Conclusion:**

The NSTC emerges as a potentially efficacious and safe treatment option for IS. NSTC could improve neurological function in different period of IS, and it has certain clinical value in secondary prevention. As a result of the poor quality and heterogeneity of the included trials, larger and standardized RCTs are needed to validate NSTC in IS treatment.

**Systematic Review Registration:**

https://www.crd.york.ac.uk/PROSPERO/display_record.php?RecordID=482981, identifier CRD42023482981.

## 1 Introduction

Ischemic stroke (IS) morbidity continually increases, causing a huge burden to individuals and the society (Saini et al., 2021). Statistical results showed that the morbidity of IS in China is 1,256/100,000 individuals (Feigin et al., 2021). It has been confirmed that IS cause a series of pathophysiological changes, including excitatory toxicity, oxidative stress, blood–brain barrier permeability, and inflammatory reactions, ultimately leading to neuron necrosis and apoptosis (Feske, 2021). At present, antithrombotic therapy, intensive lipid-lowering therapy, and antihypertensive therapy are widely implemented in clinical care and recommended as important secondary prevention strategies by stroke guidelines in different countries (Kleindorfer et al., 2021; Li et al., 2022; Dawson et al., 2022). The role of neuroprotective agents in clinical practice has received increasing attention with the continuous research on the pathological mechanisms of IS. Regrettably, despite the promising efficacy demonstrated in animal studies, many antioxidants have not shown statistically significant differences in effectiveness between intervention and control groups in clinical trials (Goenka et al., 2019). To sum up, there is a lack of accepted treatments to combine treatment and prevention. Thus, it is worthy to figure out a new therapy taking effect in both the acute and recovery periods of IS.

The Naoshuantong capsule (NSTC) is a Chinese patent medicine based on the theory of stroke etiology and pathogenesis which is “toxin damaging brain collaterals” for detail. This theory was innovated by Yongyan Wang, an academician of the Academician of the Chinese Academy of Engineering. It has been approved to treat acute and convalescent IS in China by the State Food and Drug Administration since 2001. The NSTC is composed of five Chinese herbs, which are Typhae Pollen (Pu Huang, the dry pollen of *Typha angustifolia* L.), Paeoniae Radix Rubra (Chi Shao, the root of *Paeonia lactiflora* Pall*.*), Curcumae Radix (Yu Jin, the root of *Curcuma wenyujin* Y. H. Chen et C. Ling), Gastrodiae Rhizoma (Tian Ma, the root of *Gastrodia elata* B1.), and Rhapontici Radix [Lou Lu, the radix of *Rhaponticum uniflorum* (L.) *DC*.]. Detailed information is given in Supplementary Material Section I. Research in network pharmacology has revealed that the NSTC exhibits preventive and protective effects against ischemic stroke. This is attributed to the anti-inflammatory properties of its active ingredients and their role in enhancing the expression of TGF-β1 levels, which contribute to the repair of ischemic brain tissue (Luo et al., 2020). A trial of quality of life after 6 months of follow-up in patients with IS showed that those taking NSTC in combination with aspirin recovered better in self-care and daily work ability than those taking aspirin alone (Ye et al., 2015). Furthermore, meta-analysis studies showed that the curative effect of the NSTC and chemical medicine for acute IS was significantly better than that of chemical medicine alone (Zhang et al., 2019; Chang et al., 2019).

After reviewing the guidelines on integrated traditional Chinese and modern medicine for IS (Gao et al., 2023), the shortcomings of existing studies were identified: is there a drug that can be effective in both the acute and non-acute periods of IS? On the other hand, published meta-analyses of the NSTC focused on the efficacy analysis of the NSTC in the acute phase of cerebral infarction. Thus, it is necessary to accomplish a new meta-analysis and update clinical evidence of the NSTC.

## 2 Methods

This meta-analysis was conducted in accordance with the guidelines of the Preferred Reporting Items for Systematic Reviews and Meta-Analyses (PRISMA) statement (Page et al., 2021) (Supplementary Table S2). Additionally, registration was completed in PROSPERO (CRD42023482981).

### 2.1 Search strategy

Two authors (CQ and YW) independently searched PubMed, Embase, Web of Science, Cochrane Library, China National Knowledge Internet (CNKI), Wanfang Data, China Science and Technology Journal Database (VIP), and Chinese Biomedical Literature Service System (SINOMED) databases from inception up to 1 May 2024. We used both Medical Subject Heading (MeSH) terms and free-text keywords for our search. The comprehensive search strategies are outlined in Supplementary Table S3. Study eligibility was determined through the participants, interventions, comparators, outcomes, and study design (PICOS) approach.

### 2.2 Eligibility criteria

The inclusion criteria are as follows: 1) adult patients diagnosed according to guidelines with IS; 2) the intervention must be a combination of basic treatment and NSTC; 3) comparing a placebo, standard care, neuroprotective drugs, or other ongoing interventions; 4) the outcome includes at least one of the following: i) the National Institute of Health Stroke Scale (NIHSS) for measuring neurological impairment; ii) Barthel Index (BI), modified Barthel Index (MBI), or modified Rankin Scale (mRS) used to evaluate daily life abilities; iii) Stroke-specific Quality of life (SS-QOL) for appraising the quality of life; and iv) recurrence of cerebrovascular events or other adverse events (AEs) used for safety assessment. When analyzing data, investigators plan to choose the NIHSS as the primary outcome for studies involving patients with acute IS as the research population, while mRS will be identified as the primary outcome for trials targeting non-acute period patients; and (5) study designed in the randomized controlled trial (RCT).

The gray literature was excluded because of its uncommon influence on meta-analytic findings (Hartling et al., 2017). Other excluded categories include 1) duplicate publication; 2) reviews, case reports, conference abstracts, animal experiments, correspondences, and expert insights; and 3) inadequate outcomes for meta-analysis.

### 2.3 Study selection

Abstract and full-text screening procedures were conducted simultaneously by two researchers (CQ and YW), with any discrepancies resolved through discussion. The corresponding author (XD) would be consulted when disagreements had not been resolved.

### 2.4 Data extraction

Four researchers (CQ, YW, GY, and CZ) independently extracted the following data from each trial using a standardized spreadsheet: writers, research site, number of participants, percentage of females, age range, kind of intervention, number of weeks of intervention, and results.

### 2.5 Risk of bias and quality assessment

Two investigators (GY and CZ) independently evaluated the methodological quality of the eligible studies using the standards listed in the Cochrane Risk of Bias 2 (RoB 2) (Sterne et al., 2019). RoB 2 assesses the risk of bias from the randomization process, deviations from intended interventions, missing outcome data, measurement of the outcome, and in the selection of the reported result. Each domain was classified as a low risk of bias, high risk of bias, or some concerns. For each outcome, an overall summary RoB was derived; and an overall RoB for each study was determined based on the highest RoB level in any of the domains. Disagreements between the reviewers were resolved by discussion or arbitrated with a third coauthor (YG). Furthermore, we performed Egger’s statistical tests and visually inspected the funnel plot symmetry to identify potential publication bias.

### 2.6 Certainty of evidence

Grading of evidence is such a fundamental step for a meta-analysis. Two researchers (CQ and GY) applied the Grading of Recommendations, Assessment, Development, and Evaluation (GRADE) framework (Balshem et al., 2011) to categorize evidence as “very low,” “low,” “moderate,” or “high.” Disagreements were resolved by the corresponding author (XD).

### 2.7 Statistical analysis

The study used RevMan 5.3 and Stata 14.0 software applications for analysis. Relative risk (RR) and 95% confidence interval (CI) were used as the effect size for count data such as the recurrence rate of cerebrovascular events. Measurement data such as the NIHSS score were expressed as the mean difference (MD) and 95% CI. The selection of the data pooling model hinges on the presence and magnitude of heterogeneity. When the *I* ^2^ statistics were <50%, indicating acceptable heterogeneity, the fixed-effects model was used. The random-effects model will be selected if the *I* ^2^ statistics are ≥50%, indicating significant statistical heterogeneity. The random-effects model was also utilized when conducting subgroup analysis and when notable heterogeneity among the studies was apparent. Additionally, a funnel plot was used to investigate potential publication bias if 10 or more trials were included in the meta-analysis.

## 3 Results

### 3.1 Literature search

**FIGURE 1:**
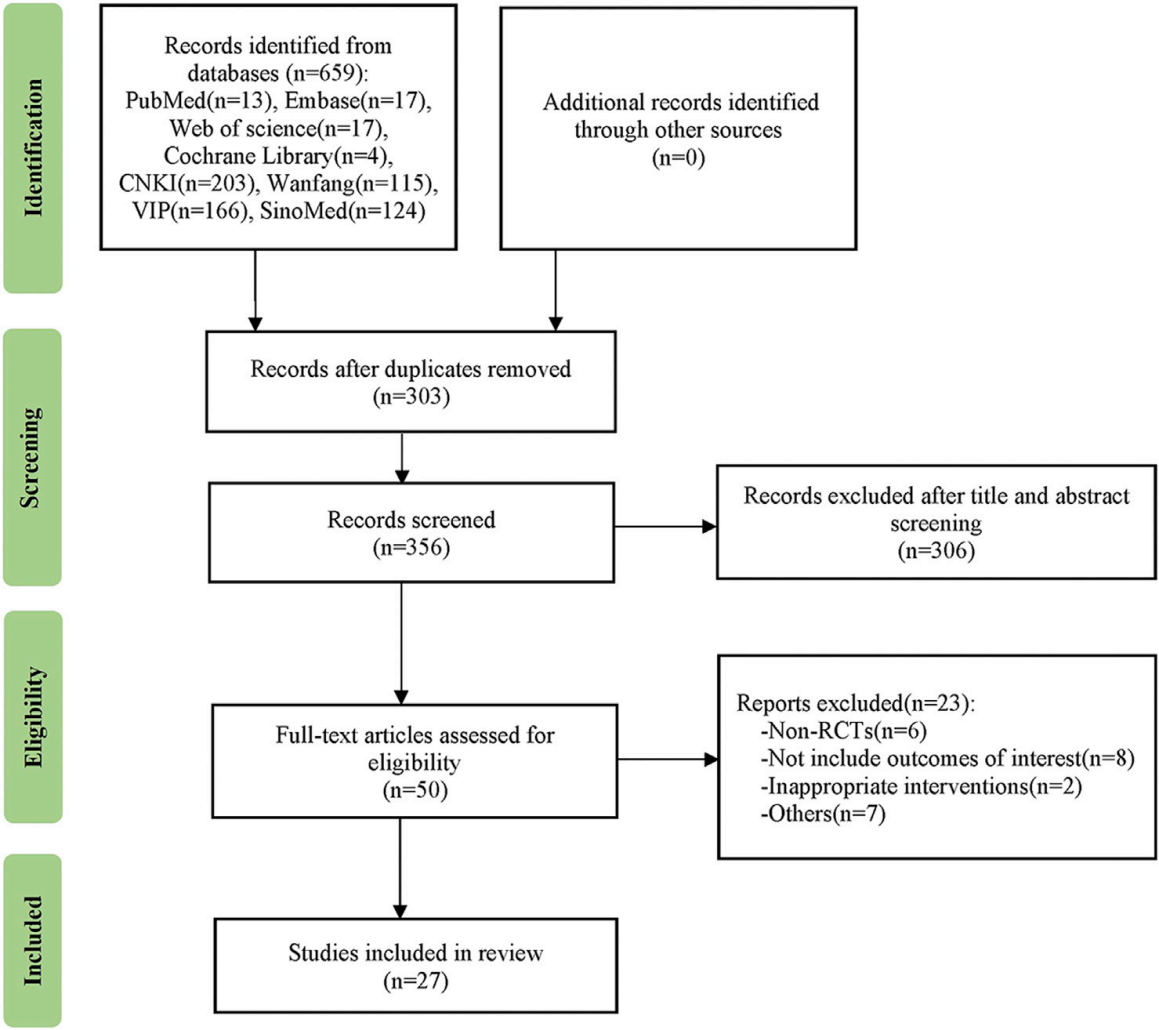
Flow diagram of study selection.

During our literature screening, a total of 659 eligible studies were initially identified in the databases. Following the removal of 303 duplicate entries, we conducted additional screening, eliminating 306 studies based on title and abstract assessment. Subsequently, a total of 23 studies were excluded due to criteria such as non-RCTs, outcomes not aligned with our interests, intervention methods inconsistent with review requirements, or other factors. Ultimately, a total of 27 studies were deemed suitable for inclusion and underwent pooled analysis. The flowchart of the literature screening process is shown in Figure 1.

### 3.2 Characteristics of the included studies

We encompassed 27 trials, enrolling a combined total of 3,139 patients. The maximum sample size in the NSTC group was up to 344, while it reached 352 in the control group. The minimum sample size was recorded at 15 in both groups. Participant ages across the studies ranged from 46 to 78 years on average. All selected articles were written in Chinese, published from 2010 to 2023. There were 15 trials including patients with acute IS, 4 trials including patients with convalescent IS, 2 trials including patients with sequelae IS, and 6 trials including patients with acute and convalescent IS. The duration of treatment ranged from 14 to 180 days. Twenty-four trials documented combination therapy alongside NSTC. Neurological impairment was evaluated by the NIHSS scale in 18 studies. Activity of daily life was assessed by the mRS in 3 studies, BI in 11 studies, and mBI in 6 studies. The quality of life was assessed by SS-QOL in two studies. Three studies mentioned the recurrence rate of cerebrovascular events, and 10 studies covered the safety outcome assessments about adverse events. The baseline characteristics of all included studies are shown in Table 1.

**TABLE 1:** Characteristics of included studies in the final meta-analysis.

Study ID	Period	Sample size		Gender (male/female)		Average age		Intervention		Duration	Outcome
		T	C	T	C	T	C	T	C		
Chen et al. (2010)	Acute	60	65	39/21	43/22	67.78 ± 19.34	65.97 ± 17.76	NSTC 0.8 g Tid po + aspirin + atorvastatin	Aspirin + atorvastatin	90 d	BI
Chen et al. (2021)	Non-acute	15	15	9/6	10/5	57.56 ± 2.19	57.4 ± 2.22	NSTC 1.2 g Tid po + aspirin + atorvastatin	Aspirin + atorvastatin	90 d	NIHSS, BI, and AE
Fu et al. (2021)	Acute	52	52	29/23	27/25	63.19 ± 7.84	64.11 ± 7.42	NSTC 1.2 g Tid po + butylphthalide	Butylphthalide	14 d	NIHSS and AE
Huang et al. (2014)	Acute	49	45	29/20	27/18	68.7 ± 6.5	68.5 ± 6.3	NSTC 1.2 g Tid po + aspirin + lipitor + edaravone	Aspirin + lipitor + edaravone	84 d	NIHSS and BI
Huang (2015)	Acute	30	30	20/10	18/12	63.6 ± 3.0	62.3 ± 2.5	NSTC 1.2 g Tid po + aspirin + atorvastatin	Aspirin + atorvastatin	90 d	BI
Huang et al. (2020)	Acute	75	75	40/35	42/33	55.43 ± 9.18	54.94 ± 7.81	NSTC 1.2 g Tid po + yurekline	Yurekline	14 d	NIHSS and AE
Li (2011)	Non-acute	33	33	42/24	42/24	46–78	46–78	NSTC 0.8 g Tid po + aspirin	Aspirin	30 d	mBI
Li et al. (2020)	Acute	44	44	25/19	27/17	56.33 ± 9.11	56.28 ± 9.14	NSTC 1.2 g Tid po + yurekline	Yurekline	21 d	NIHSS and AE
Li and Wang (2020)	Non-acute	30	30	16/14	17/13	57.48 ± 7.59	56.32 ± 8.03	NSTC 1.2 g Tid po + rehabilitation training therapy	Rehabilitation training therapy	28 d	NIHSS
Lin et al. (2010)	Non-acute	22	23	14/8	15/8	65.96 ± 4.78	63.76 ± 5.23	NSTC 0.8 g Tid po + aspirin	Placebo + aspirin	30 d	mBI
Lin et al. (2011)	Non-acute	22	22	14/8	12/10	65.96 ± 4.78	66.83 ± 4.65	NSTC 0.8 g Tid po + aspirin	Basic treatment	30 d	mBI
Lin et al. (2023)	Acute	60	60	24/36	22/38	72.31 ± 6.52	71.74 ± 5.65	NSTC 1.2 g Tid po + butylphthalide	Butylphthalide	14 d	NIHSS, BI
Liu and Li (2012)	Acute	87	86	52/35	53/33	69.0 ± 5.04	68.2 ± 5.05	NSTC 1.2 g Tid po + basic treatment	Basic treatment	90 d	NIHSS, BI, and recurrence rate of cerebrovascular events
Liu and Xu (2018)	Acute	82	80	45/37	42/38	63.36 ± 9.18	62.68 ± 9.12	NSTC 1.2 g Tid po + basic treatment	Basic treatment	28 d	NIHSS
Lu et al. (2020)	Acute	33	33	21/12	20/13	61.60 ± 5.15	61.56 ± 4.89	NSTC 1.2 g Tid po + butylphthalide	Butylphthalide	28 d	NIHSS, BI, and AE
Luo (2022)	Non-acute	50	50	35/15	30/20	54.48 ± 5.29	54.35 ± 5.26	NSTC 1.2 g Tid po + clopidogrel	Clopidogrel	60 d	NIHSS, BI, and AE
Ma and Lan (2011)	Non-acute	35	30	19/11	22/13	60.1 ± 7.2	59.8 ± 8.3	NSTC 1.2 g Tid po + citicoline	Citicoline	28 d	mBI
Pan (2013)	Acute	69	66	35/31	39/30	63.5 ± 15.9	62.5 ± 16.7	NSTC 1.2 g Tid po + aspirin + atorvastatin	Aspirin + atorvastatin	28 d	BI and recurrence rate of cerebrovascular events
Peng et al. (2012)	Non-acute	45	45	22/23	26/19	60.26 ± 9.52	61.75 ± 10.26	NSTC 1.2 g Tid po	Blank control	180 d	NIHSS
Qiao and Li (2021)	Acute	51	51	32/19	31/20	62.34 ± 6.56	61.53 ± 7.38	NSTC 1.2 g Tid po + butylphthalide	Butylphthalide	14 d	NIHSS and BI
Qin et al. (2019)	Acute	30	30	15/15	16/14	69.48 ± 10.49	67.52 ± 9.87	NSTC 1.2 g Tid po + aspirin + atorvastatin	Aspirin + Atorvastatin	60 d	NIHSS, mRS, and mBI
Wang et al. (2015)	Non-acute	20	24	15/5	18/6	72.47 ± 11.25	69.04 ± 8.70	NSTC 1.2 g Tid po + clopidogrel	Clopidogrel	28 d	mBI, recurrence rate of cerebrovascular events, and AE
Wang et al. (2021)	Acute	45	45	26/19	24/21	65.86 ± 5.24	66.23 ± 5.32	NSTC 1.2 g Tid po + aspirin + clopidogrel + rt-PA	Aspirin + clopidogrel + rt-PA	14 d	NIHSS, mRS, and AE
Wang (2022)	Acute	64	64	36/28	38/26	61.28 ± 5.56	62.63 ± 5.48	NSTC 1.2 g Tid po + rt-PA	rt-PA	14 d	NIHSS and mRS
Wu (2021)	Acute	60	60	37/23	34/26	48.42 ± 5.74	48.67 ± 5.49	NSTC 1.2 g Tid po + butylphthalide	Butylphthalide	28 d	NIHSS and AE
Xue et al. (2022)	Acute	61	61	33/28	32/29	62.11 ± 5.86	62.23 ± 5.92	NSTC 1.2 g Tid po + butylphthalide	Butylphthalide	14 d	NIHSS, BI, and SS-QOL
Ye et al. (2015)	Non-acute	344	352	232/113	231/121	62.37 ± 9.97	62.82 ± 9.97	NSTC 1.2 g Tid po + aspirin	Aspirin	180 d	SS-QOL and AE

T, treatment group; C, control group; d, day; AE, adverse event.

### 3.3 Risk of bias assessment

We rated the overall bias as “low risk of bias” in 1 study (Fu et al., 2021), while 10 of the remaining studies were judged to be “some concerns” and 16 were judged to be “high risk of bias,” suggesting the poor quality of the selected RCTs.

**FIGURE 2:**
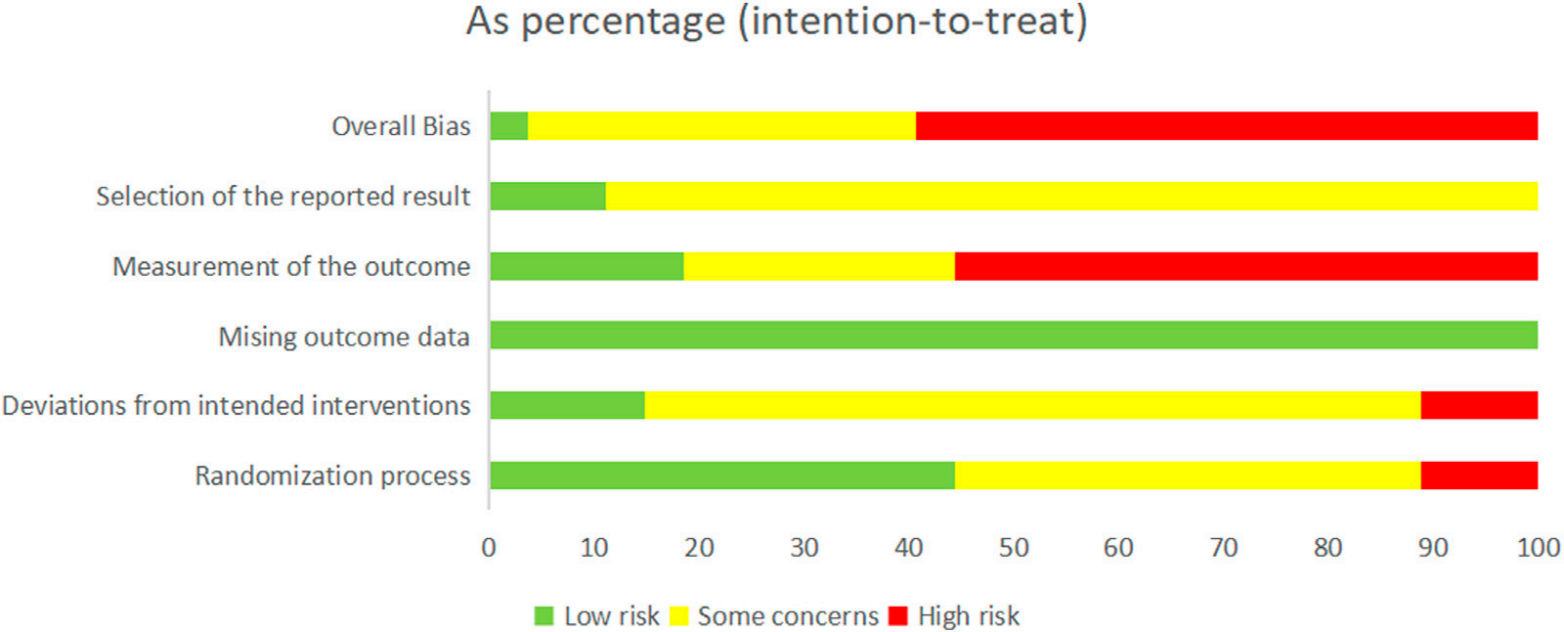
Risk of bias graph.

In three studies, the “randomization process” was assessed to have a “high risk of bias” due to the improper concealment of allocation methods. Conversely, a total of 12 studies were rated as having “some concerns” for not providing a detailed and explicit description of their random sequence despite mentioning it. Regarding bias risk due to “deviations from the intended interventions,” three studies were assessed as having a “high risk of bias” because they did not use blinding. In contrast, four studies were judged to have a “low risk of bias” due to their double-blind design and appropriate intention-to-treat analysis. Furthermore, all studies were judged to have a “low risk of bias” in terms of “missing outcome data” as no reports of missing data were found or the missing rate could be ignored, and the missing data were balanced between the experimental and control groups. Regarding the bias risk due to “measurement of the outcome,” 15 studies were assessed as having a “high risk of bias” because of inappropriate measurement methods or because there were gaps in outcome measures between groups. Regarding the bias risk due to the “selection of the reported result,” three studies were rated as having a “low risk of bias” due to their transparent observational reports, while 24 studies raised “some concerns” due to the lack of relevant reporting. In conclusion, the detailed methodological quality assessment of the 27 RCTs is summarized in Figure 2 and Supplementary Figure S1.

### 3.4 Primary outcomes

#### 3.4.1 Acute period of the NIHSS

**FIGURE 3:**
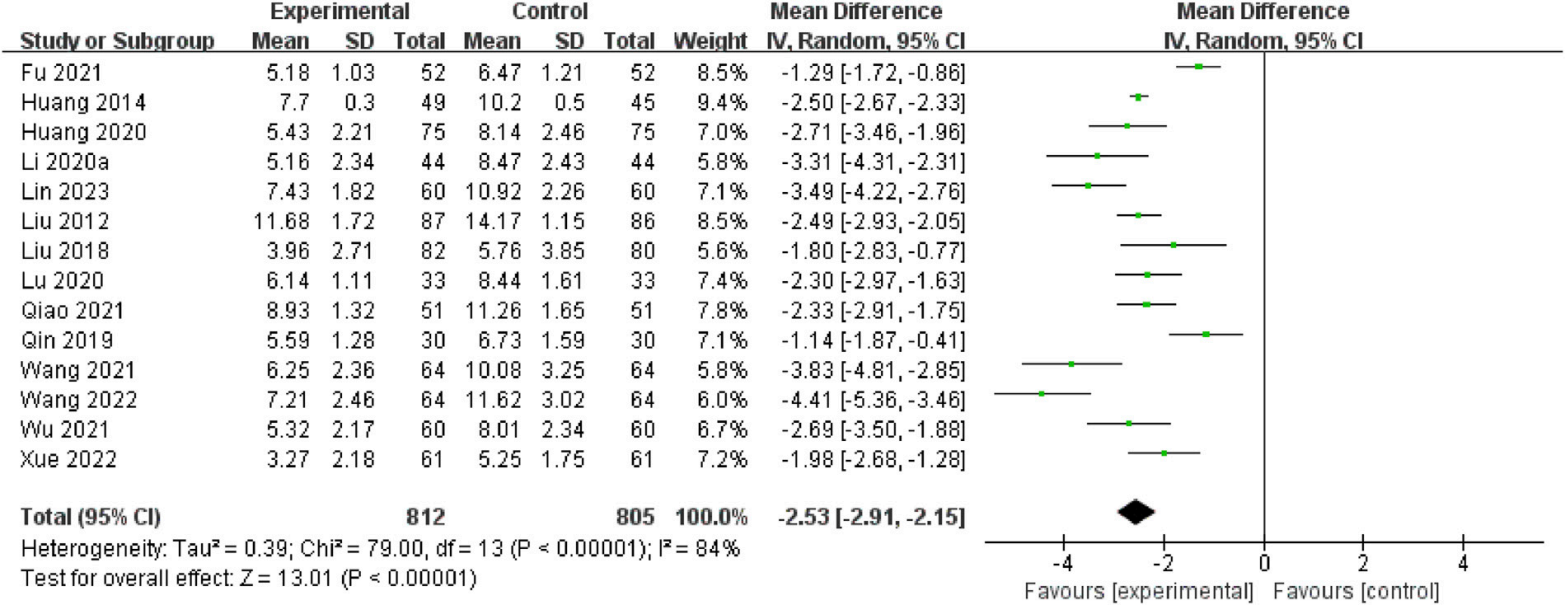
Forest plot of the acute period of the NIHSS.

A total of 14 RCTs (Lin et al., 2023; Wang, 2022; Xue et al., 2022; Qiao and Li, 2021; Wu, 2021; Wang et al., 2021; Fu et al., 2021; Li et al., 2020; Lu et al., 2020; Huang et al., 2020; Qin et al., 2019; Liu and Xu, 2018; Huang et al., 2014; Liu and Li, 2012) assessed this outcome through the acute period of the NIHSS. The pooled data demonstrated that the NSTC group exhibited a reduction in NIHSS scores during the acute period (MD = −2.53; 95% CI: −2.91, −2.15; *p* < 0.00001). Because of the substantial heterogeneity (*I* ^2^=84%, *p* < 0.00001), a random-effects model was used (Figure 3). The sensitivity analysis showed that even after deleting each study one at a time, the statistical heterogeneity was not altered (see Supplementary Figure S2). Subgroup analyses were subsequently conducted based on the intervention types (NSTC 1.2 g Tid po + butylphthalide vs butylphthalide, MD = −2.32; 95% CI: −2.95, −1.68; NSTC 1.2 g Tid po + aspirin + atorvastatin vs aspirin + atorvastatin, MD = −1.14; 95% CI: −1.87, −0.41; NSTC 1.2 g Tid po + yurekline vs yurekline, MD = −2.93; 95% CI: −3.52, −2.33) (Supplementary Figure S3). The results indicated that the NSTC combined with butylphthalide, yurekline, aspirin, and atorvastatin led to a statistically apparent reduction in NIHSS scores.

#### 3.4.2 Modified Rankin Scale at 3 months

There was no literature that both meets the eligibility criteria and uses the mRS at 3 months as the outcome. Three out of the 27 included articles used the mRS as the outcome, but they focused on 14 days or 60 days from the day of onset. Details are discussed in the following sections.

### 3.5 Secondary outcomes

#### 3.5.1 Non-acute period of the NIHSS

**FIGURE 4:**
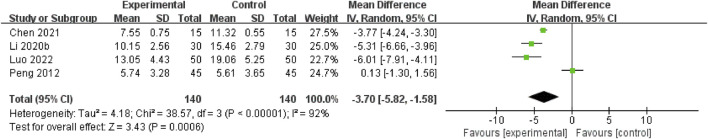
Forest plot of the non-acute period of the NIHSS.

A total of four RCTs (Luo, 2022; Chen et al., 2021; Li and Wang, 2020; Peng et al., 2012) assessed this outcome through the non-acute period of the NIHSS. The pooled data demonstrated that the NSTC group exhibited a reduction in NIHSS scores during the non-acute period (MD = −3.70; 95% CI: −5.82, −1.58; *p* = 0.0006). Because of the substantial heterogeneity (*I* ^2^=92%, *p* < 0.00001), a random-effects model was used (Figure 4). The sensitivity analysis showed that even after deleting each study one at a time, the statistical heterogeneity was not altered (see Supplementary Figure S4).

#### 3.5.2 Modified Rankin scale

**FIGURE 5:**
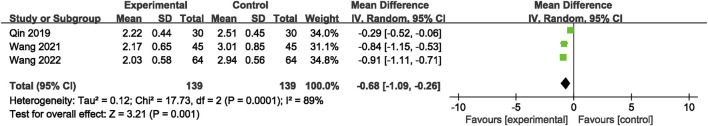
Forest plot of the mRS.

Three RCTs (Qin et al., 2019; Wang et al., 2021; Wang, 2022) reported mRS grading. The analytic results showed that adding the NSTC led to a reduction in mRS scores (MD = −0.68; 95% CI: −1.09, −0.26; *p* = 0.001) (Figure 5). A random-effects model was chosen due to significant heterogeneity (*I* ^2^=89%, *p* = 0.001). The sensitivity analysis revealed that after the study by Qin et al. (2019) was screened out, the NSTC group demonstrated superior effectiveness over the control group in terms of neurological recovery (MD=−0.89; 95% CI: −1.06, −0.72) with no heterogeneity (Supplementary Figure S5).

#### 3.5.3 Barthel Index

**FIGURE 6:**
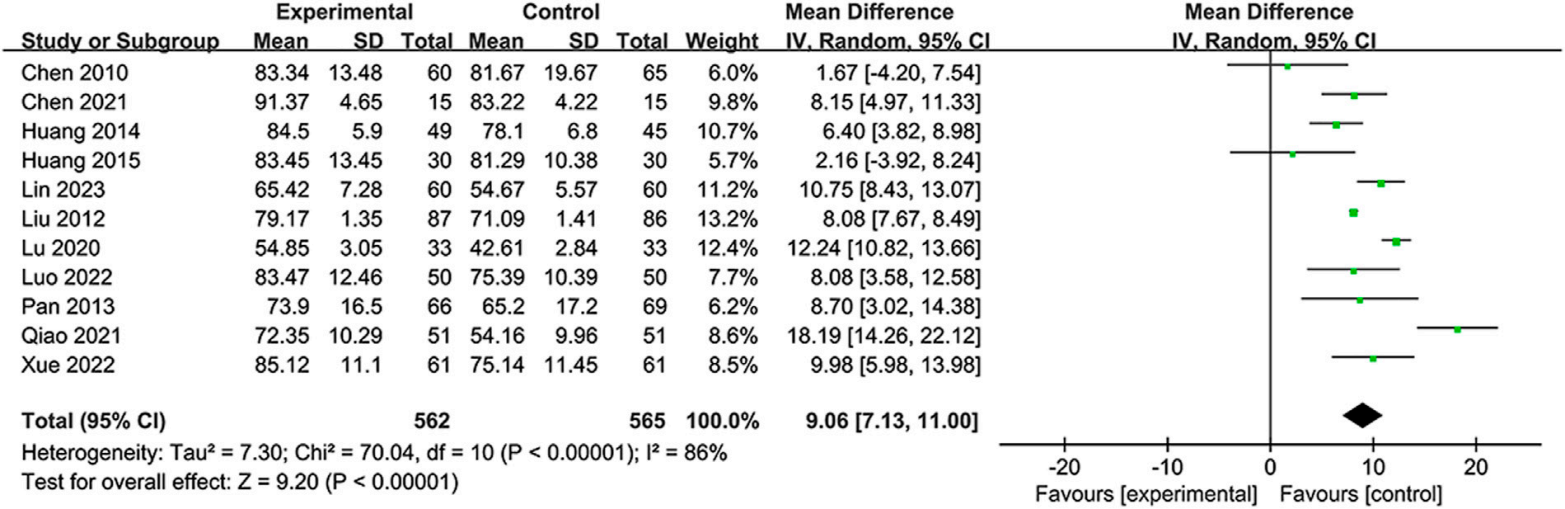
Forest plot of the BI.

A total of 11 RCTS reported BI to demonstrate the ability to perform activities of daily life (Chen et al., 2010; Liu and Li, 2012; Pan, 2013; Huang et al., 2014; Huang, 2015; Lu et al., 2020; Chen et al., 2021; Qiao and Li, 2021; Luo, 2022; Xue et al., 2022; Lin et al., 2023). Due to substantial heterogeneity in the BI (*I* ^2^ = 86%, *p* < 0.00001), the random-effects model was selected. The data (Figure 6) showed that the NSTC group showed an improved BI compared to only using conventional treatment such as aspirin or neuroprotective agents (MD = 9.06; 95% CI: 7.13, 11.00; *p* < 0.00001). Additional sensitivity analysis showed that when individual studies were sequentially excluded, statistical heterogeneity was not altered (Supplementary Figure S6). Subgroup analyses were developed focusing on the period, intervention, and duration time. The results revealed dramatic differences in mean values between the NSTC and control groups during the acute period (MD = 11.04; 95% CI: 8.26, –13.82) and non-acute period (MD = 8.13; 95% CI: 5.53, –10.72) (Supplementary Figure S7), while at the intervention, the NSTC combined with aspirin and atorvastatin or butylphthalide could improve the BI (NSTC combined with aspirin and atorvastatin vs aspirin and atorvastatin: MD = 6.86; 95% CI: 3.33, 10.39; NSTC combined with butylphthalide vs butylphthalide: MD = 12.56; 95% CI: 9.94, –15.18) (Supplementary Figure S8). By duration, the BI score was also statistically significant when the treatment course of the NSTC was 14 days, 28 days, and 90 days (14 days: MD = 12.86; 95% CI: 8.17, 17.56; 28 days: MD = 11.58; 95% CI: 8.89, 14.28; 90 days: MD = 6.34; 95% CI: 3.61, –9.08) (Supplementary Figure S9).

#### 3.5.4 Modified Barthel Index

**FIGURE 7:**
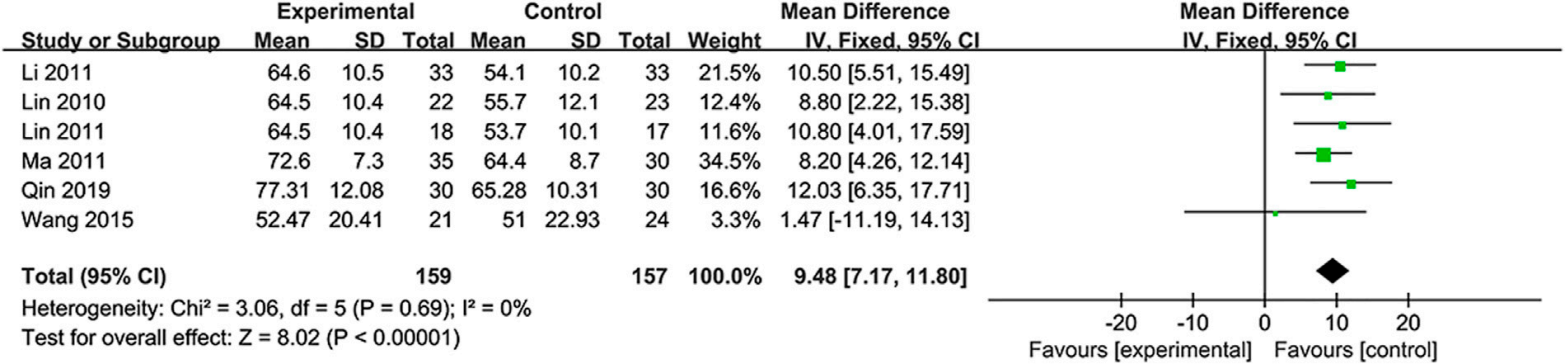
Forest plot of the mBI.

The independence was assessed by MBI scores in six RCTs (Lin et al., 2010; Li, 2011; Lin et al., 2011; Ma and Lan, 2011; Wang et al., 2015; Qin et al., 2019). Considering the absence of heterogeneity (*I* ^2^=0%, *p* = 0.69), the fixed-effects model was selected. Based on the data, the NSTC group outperformed in terms of the mBI (MD = 9.48; 95% CI: 7.17, 11.80; *p* < 0.00001) (Figure 7). Subgroup analyses were developed focusing on different treatment periods and durations. The mean differences in the mBI were significantly different between the NSTC and control groups during both the acute period (MD=11.17; 95% CI: 7.42 –14.92) and non-acute period (MD=7.61; 95% CI: 3.84, 11.37) (Supplementary Figure S110). Additionally, the mBI of the NSTC group showed improvement after 28 and 30 days of treatment, with statistically significant differences observed (MD = 8.97; 95% CI: 6.44, 11.51) (Supplementary Figure S11).

#### 3.5.5 SS-QOL

**FIGURE 8:**
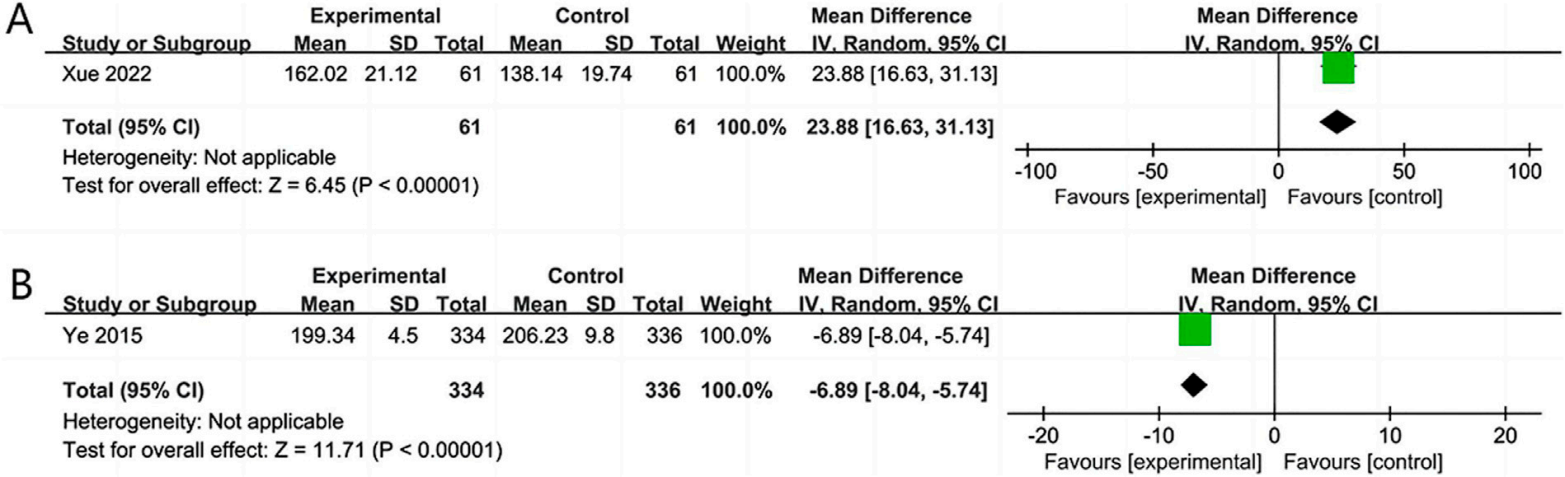
**(A)** Forest plot of SS-QOL at 14 days. **(B)** Forest plot of SS-QOL at 180 days.

Two RCTs (Ye et al., 2015; Xue et al., 2022) assessed the quality of life over 14 and 180 days. The results revealed that the NSTC group showed improvement in SS-QOL at 14 days (MD = 23.88; 95% CI: 16.63, 31.13; *p* < 0.00001). However, at 180 days, the NSTC group did not exhibit improvement in SS-QOL (MD = −6.89; 95% CI: −8.04, −5.74; *p* < 0.00001) (Figure 8).

#### 3.5.6 Recurrence rate of cerebrovascular events

**FIGURE 9:**
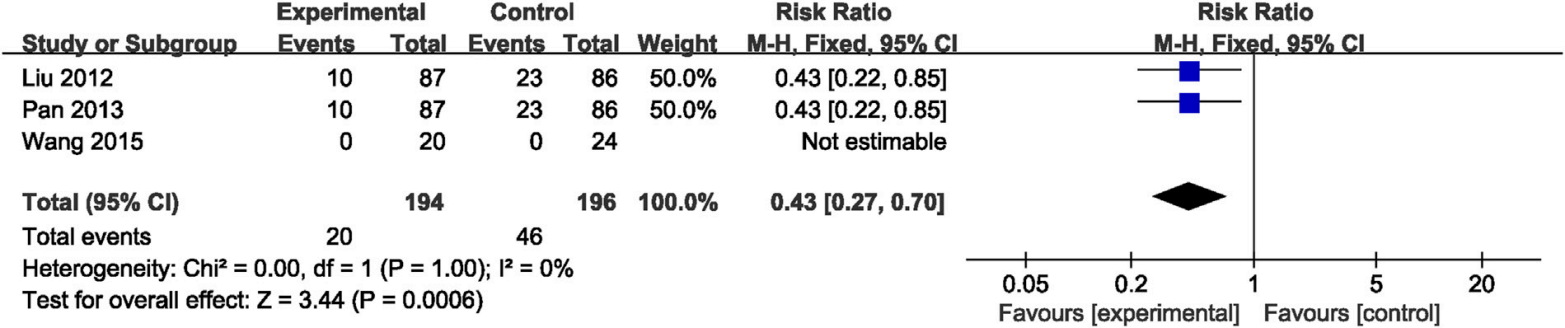
Forest plot of the recurrence rate of cerebrovascular events.

Three RCTs (Liu and Li, 2012; Pan, 2013; Wang et al., 2015) involving 390 patients reported the recurrence rate of cerebrovascular events, revealing a great difference between the NSTC and control groups (RR = 0.43; 95% CI: 0.27, 0.70; *p* = 0.0006; *I* ^2^ = 0%) (Figure 9). Thus, there is evidence safely suggesting that the NSTC may reduce the recurrence rate of cerebrovascular events.

#### 3.5.7 Adverse events

**FIGURE 10:**
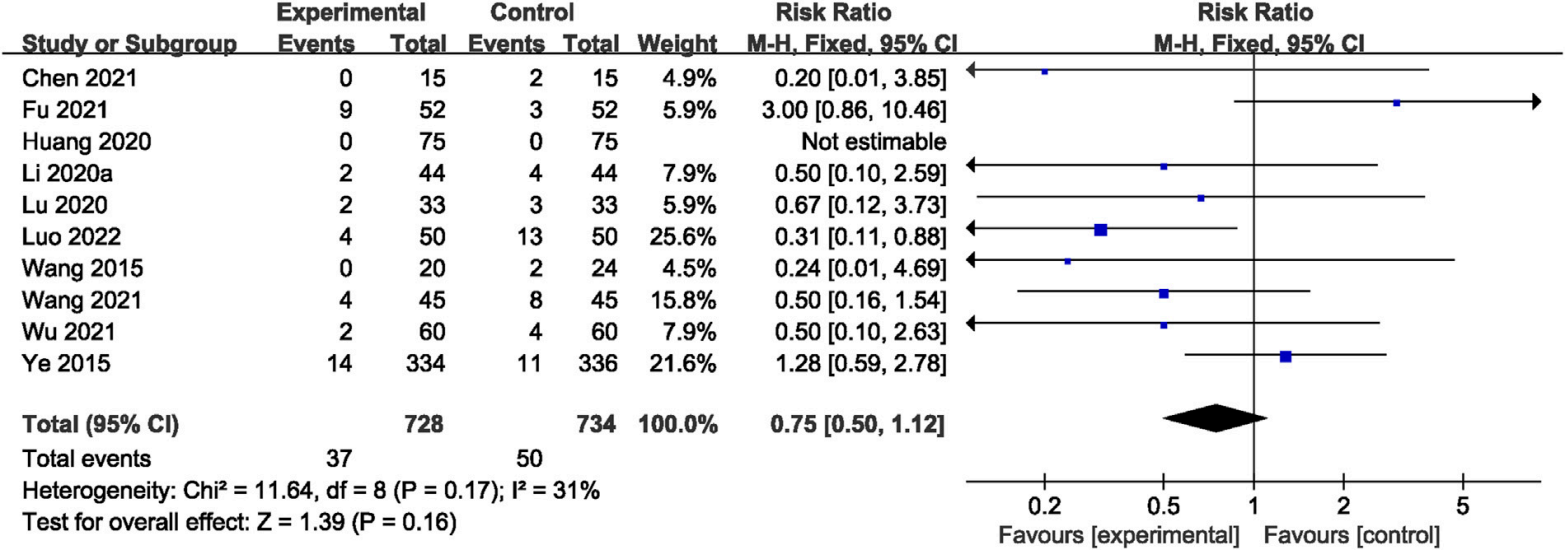
Forest plot of the AE.

Across all studies, no related AEs were reported. A total of 10 RCTs (Ye et al., 2015; Wang et al., 2015; Li et al., 2020; Lu et al., 2020; Huang et al., 2020; Chen et al., 2021; Wu, 2021; Wang et al., 2021; Fu et al., 2021; Luo, 2022) reported adverse reactions. However, there was no significant difference observed between the two groups (RR = 0.75; 95% CI: 0.50, 1.12; *p* = 0.16; *I* ^2^ = 31%) (Figure 10). The most frequently reported adverse reactions were nausea, vomiting, abnormal liver function, gastrointestinal reaction, and skin rash. Specific adverse events are given in Supplementary Table S4.

### 3.6 Publication bias

**FIGURE 11:**
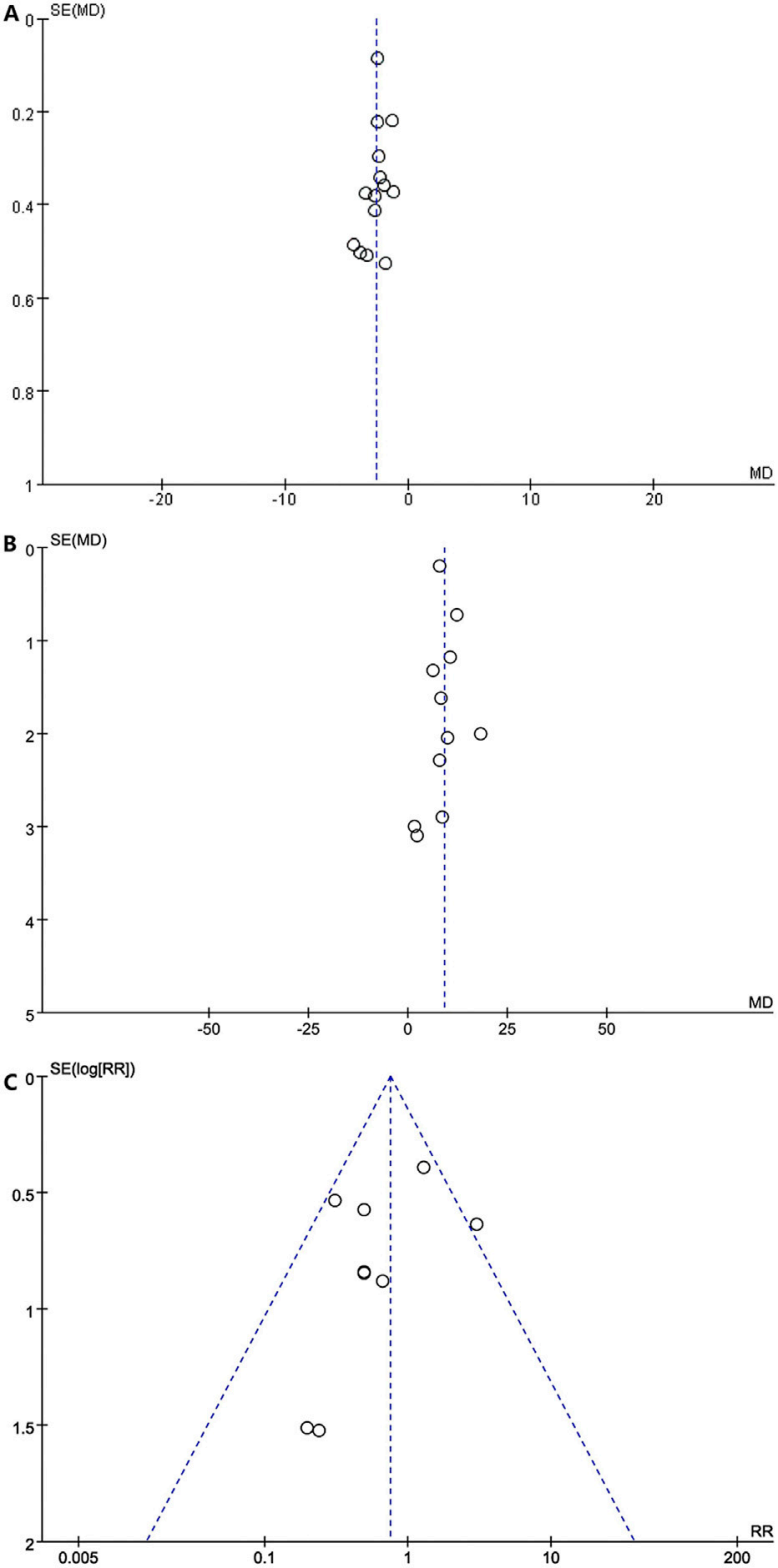
**(A)** Funnel plots of the NlHSS (acute period). **(B)** Funnel plots of the Bl. **(C)** Funnel plots of the AE.

The visual inspection of funnel plots [Figure 11A shows the funnel plots of the NIHSS (acute period), Figure 11B shows the funnel plots of the BI, and Figure 11C shows the funnel plots of AEs] and statistical tests identified substantial symmetry and revealed no significant publication bias among the involved trials concerning the acute period of the NIHSS score (Egger’s test, *p* = 0.303; Supplementary Figure S12), the BI score (Egger’s test, *p* = 0.448, Supplementary Figure S13), and adverse events (Egger’s test, *p* = 0.510, Supplementary Figure S14). The publication bias of the mRS, mBI, recurrence rate of cerebrovascular events, and SS-QOL score could not be estimated for only less than 10 RCTs.

### 3.7 Certainty of evidence

The certainty of evidence regarding the impact of the NSTC on adverse events was rated “moderate;” for the NIHSS (acute period), BI, mBI, SS-QOL, and the recurrence rate of cerebrovascular events, it was rated as “low;” and for the NIHSS (non-acute period) and mRS score, it was rated “very low” (refer to Table 2). The quality of evidence was assessed from moderate to very low, primarily because of poor methodological quality, significant inconsistency, and limited sample size.

**TABLE 2:** GRADE evidence profiles.

Outcome	No. of participants (no. of studies)	Certainty assessment						Relative effect (95% CI)	Absolute effect (95% CI)	Certainty
		Study design	Risk of bias	Inconsistency	Indirectness	Imprecision	Other considerations			
NIHSS (acute period)	1,617 (14)	High	Serious^a^	Serious^b^	Not serious	Not serious	None	—	MD 2.53 lower (2.91–2.15 lower)	⊕⊕○○ Low
NIHSS (non-acute period)	280 (4)	High	Serious^a^	Serious^b^	Not serious	Serious^c^	None	—	MD 3.70 lower (5.82–1.58 lower)	⊕○○○ Very low
mRS	278 (3)	High	Serious^a^	Serious^b^	Not serious	Serious^c^	None	—	MD 0.68 lower (1.09–0.26 lower)	⊕○○○ Very low
BI	1,127 (11)	High	Serious^a^	Serious^b^	Not serious	Not serious	None	—	MD 5.51 higher (2.30–8.72 higher)	⊕⊕○○ Low
mBI	316 (6)	High	Serious^a^	Not serious	Not serious	Serious^c^	None	—	MD 9.48 higher (7.17–11.8 higher)	⊕⊕○○ Low
SS-QOL	792 (2)	High	Serious^a^	Serious^b^	Not serious	Not serious	None	—	MD 8.28 higher (21.87 lower-38.43 higher)	⊕⊕○○ Low
Recurrence rate of cerebrovascular events	390 (3)	High	Serious^a^	Not serious	Not serious	Serious^c^	None	RR 0.43 (0.27–0.70)	—	⊕⊕○○ Low
AE	1,462 (10)	High	Serious^a^	Not serious	Not serious	Not serious	None	RR 0.75 (0.50–1.12)	—	⊕⊕⊕○ Moderate

^a^ Poor methodological quality.

^b^ Serious inconsistency (*I* ^2^ ≥ 50%).

^c^ The sample size is not sufficient (n<400).

## 4 Discussion

### 4.1 Summary of the findings

To the best of our knowledge, this study represents the first systematic review and meta-analysis to innovatively pay more attention to the clinical efficacy of the NSTC in the acute and non-acute phases of IS patients. The staging of stroke is a key problem in the daily practice of neurology. In addition, this study also focused on the safety of the NSTC in secondary prevention. Following the purpose, the study encompassed 27 RCTs involving 3,139 participants, suggesting potential improvements in the BI and mBI alongside reductions in the NIHSS, mRS scores, and the risk of recurrent cerebrovascular events. NSTC treatment demonstrated enhancements in SS-QOL at the 14-day mark. However, over the extended 180-day period, the patients’ quality of life decreased. Notably, there was no increase in adverse reactions. NSTC therapy emerges as a safe and effective approach, potentially valuable for the secondary prevention of IS.

Regarding neurological deficits, the findings suggest that incorporating NSTC prescriptions may accelerate the recovery process of neurological function in IS patients, as assessed by the NIHSS. Additionally, when combined with yurekline and butylphthalide capsules, the NSTC demonstrated a significant reduction in NIHSS scores. Three RCTs indicated that the NSTC could depress the disability rate assessed by the mRS. Moreover, treatment with the NSTC significantly improved the activities of daily life, as reflected in the BI and mBI scores. The subgroup analysis of BI scores revealed a significant difference between the NSTC and control groups in both acute and non-acute stages of stroke. NSTC combined with aspirin and atorvastatin calcium tablets or butylphthalide demonstrated improvements in BI scores, with statistically significant differences observed. Furthermore, compared to the control group, the NSTC group exhibited significant BI score improvements at 14, 28, and 90 days of treatment. In the subgroup analysis of mBI scores, the NSTC group played a better role in both acute and non-acute periods of IS, with statistically significant differences noted. Additionally, the mBI scores in the NSTC group improved after 28 and 30 days of treatment, with statistically significant differences observed. The results of the two RCTs indicated that adding NSTC prescriptions improved patient SS-QOL scores at 14 days. However, no improvement was observed in the NSTC group compared to the control group at 180 days. This may be due to a lack of rigorous design, resulting in insufficient sample size and bias in the results. On the other hand, it suggests that we may need more long-term follow-up studies for further evaluation. The NSTC group exhibited a statistically significant reduction in the recurrence rate of cerebrovascular events compared to the control group. Among the 27 including RCTs, 6 reported the incidence of adverse effects and clinical features, with no significant distinctions observed between groups.

### 4.2 Possible therapeutic mechanisms of the NSTC

**FIGURE 12:**
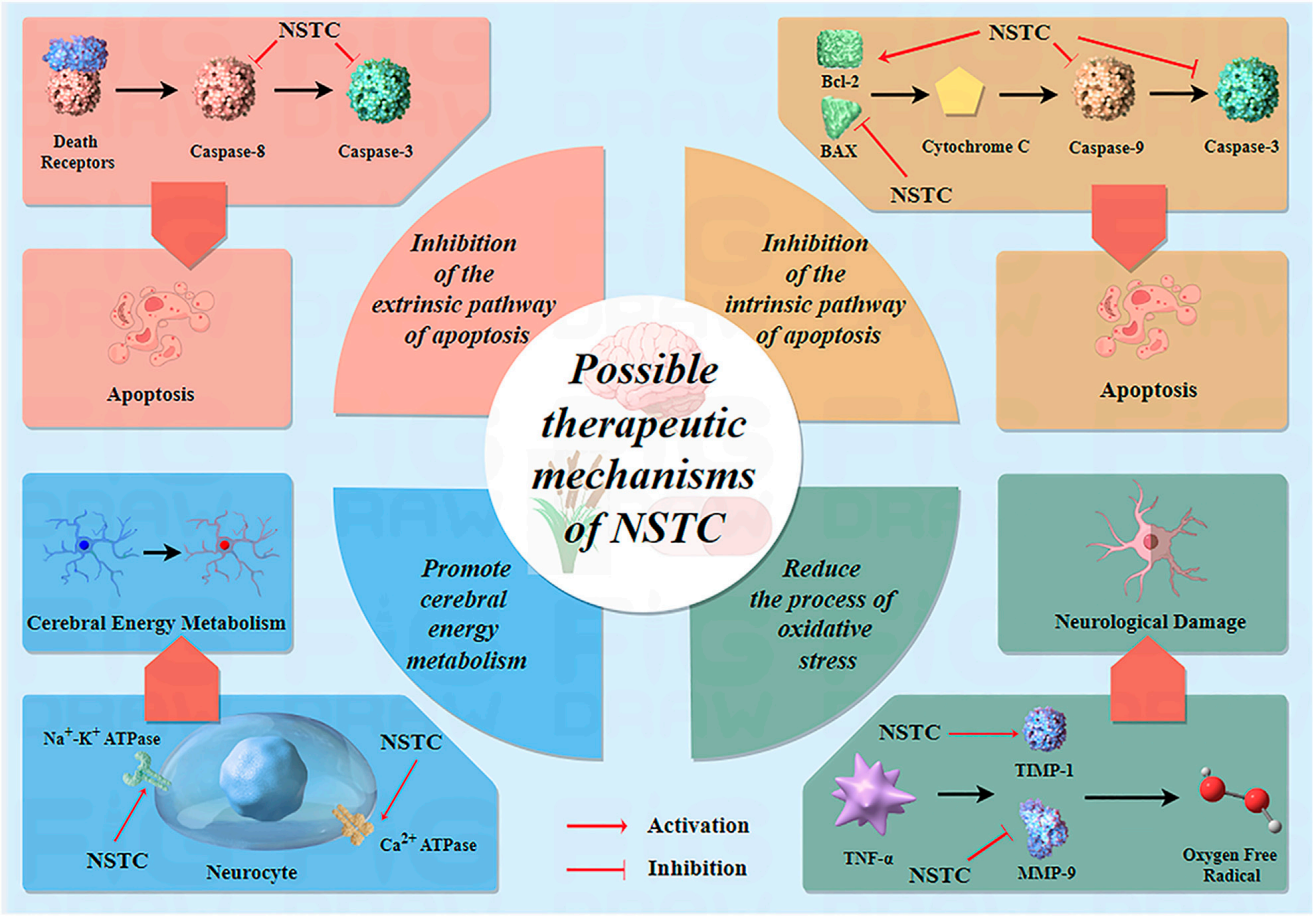
Potential mechanism of the NSTC.

Over the past 15 years, numerous experimental studies on NSTC treatment for IS have been published globally, revealing its mechanisms of action at different levels, including at the molecule, cell, tissue, and organ levels. These studies have provided evidence of its effectiveness. In the earlier literature dating back to 2010, Xiang et al. (2010) conducted a study on focal cerebral ischemia in rats and discovered that the NSTC significantly decreased the area of cerebral infarction, alleviated neurological functional damage, and reduced neuronal apoptosis. The results of this basic research further support the results of the meta-analysis of this study, and NSTC can improve the degree of neurological deficit in patients with acute and non-acute IS in clinical practice. In addition, Xiang et al. (2010) and Huang et al. (2015) found that the NSTC could inhibit the overexpression of Bax and caspase-3 and enhance the production of Bcl-2. These results indicate that the NSTC can inhibit neuronal apoptosis and has a neuroprotective effect on cerebral ischemia. Furthermore, at the molecular level, experimental investigations by Liu et al. (2014) proved that the NSTC enhanced the activity of Na^+^-K^+^ ATPase and Ca^2+^ ATPase, reduced lactic acid levels in brain tissues, and ultimately facilitated brain tissue metabolism. At the genetic level, Zheng et al. (2020) proposed that the NSTC could apparently reduce the inflammatory factors, improve the expression of MMP-9 and TIMP-1, and decrease its oxidative stress index in a rat model of acute cerebral infarction. Recently, using experimental observations in rats (cerebral ischemia/reperfusion injury), Guo et al. (2022) found that the NSTC could significantly increase the expression level of the vascular endothelial growth factor and basic fibroblast growth factor, significantly increase the amount of angiogenesis during cerebral ischemia and reperfusion, and also significantly lessen the area of cerebral infarction during cerebral ischemia and reperfusion. Angiogenesis is essential for the recovery of ischemic areas. In clinical patients, angiogenesis not only contributes to short-term recovery but also helps improve long-term outcomes, including reduced recurrence rates. This also supports the results of our meta-analysis. More specific details of related studies on the mechanisms of NSTC treatment are shown in Table 3. Based on all evidence above, we can safely draw a conclusion that the NSTC has a significant therapeutic effect and improvement effect on the brain injury caused by IS. More specific mechanisms of drug action of the NSTC are given in Figure 12.

**TABLE 3:** Possible therapeutic mechanisms of the NSTC.

Published year	Title	Potential mechanisms	Experimental models used
Xiang et al. (2010)	Chinese medicine Naoshuantong attenuates cerebral ischemic injury by inhibiting apoptosis in a rat model of stroke	(1) Significantly reduced the cerebral infarct area, attenuated neurological functional deficits, and reduced neuronal apoptosis in the ischemic cortex and in the CM region of the hippocampus. (2) Suppressed the overexpression of Bax and activated caspases-3, -8, and -9 and also inhibited the reduction in Bcl-2 expression and markedly depressed the Bax/Bcl-2 ratio.	Rats, middle cerebral artery occlusion (MCAO)
Liu et al. (2014)	Protective effects of traditional Chinese medicine formula Naoshuantong capsule on hemorheology and cerebral energy metabolism disorders in rats with blood stasis	Improved Na^+^-K^+^ adenosine triphosphatase (ATPase) and Ca^2+^ ATPase activity, as well as lowered the lactic acid level in brain tissues	Rats, blood stasis
Huang et al. (2015)	Herbal compound Naoshuantong capsule attenuates retinal injury in the ischemia/reperfusion rat model by inhibiting apoptosis	Attenuated the changes and damage to the ischemic retina in the rat model, inhibited the overexpression of Bax and caspase-3, and increased the expression of Bcl-2	Rats, ischemic ophthalmopathy
Liu et al. (2018)	Discovery of bioactive compounds in the Chinese herbal formula Naoshuantong capsule (NSTC) against hemorheological disorders	(1) Isorhamnetin-3-O-neohesperidoside had impacts on erythrocyte aggregation, erythrocyte deformability, and platelet aggregation. (2) Benzoyl-oxypaeoniflorin was associated with erythrocyte aggregation, coagulation function, and platelet aggregation	Rats, acute blood stasis
Guo et al. (2022)	Effect of the Naoshuantong capsule on angiogenesis in rats with focal cerebral ischemia and reperfusion	Increased the expression levels of the vascular endothelial growth factor and basic fibroblast growth factor in rats with cerebral ischemia and reperfusion	Rats, cerebral ischemia/reperfusion injury
Zheng et al. (2020)	Effect of the Naoshuantong capsule on the expression of inflammatory factors, MMP-9, and TIMP-1 and oxidative stress indicators in the rat model of acute cerebral infarction	Effectively reduced the level of inflammatory factors, reduced MMP-9 and improved TIMP-1 expression, and reduced their oxidative stress indicators in the rat model of acute cerebral infarction	Rats, cerebral ischemia/reperfusion injury

### 4.3 Strengths and limitations

This study focuses on the efficacy and safety of the NSTC for different stages of ischemic stroke. In contrast, previous studies (Chang et al., 2019) were merely focused on patients in the acute period. Considering that the NSTC is a kind of patent medicine based on toxins damaging brain collaterals theory in traditional Chinese medicine, which emphasizes the entire process of the disease, we included studies on stroke in the acute period, recovery period, and sequelae period in this study. The analytic results showed that using the NSTC could enhance the neurological function of patients. Furthermore, the meta-analysis showed that the NSTC could reduce the recurrence of cerebrovascular events. Lastly, our study produced the GRADE evidence-level evaluation, which is conducive to the revision of guidelines and consensus for better service and can also guide clinical practice. The NSTC might, therefore, prove to be an effective therapy choice in the future. Moreover, it is hoped that this systematic review can provide some assistance to future research.

However, this meta-analysis exhibited several shortcomings. First, certain RCTs exhibited a lower methodological grade as they were not able to adequately elucidate the procedures in randomizing, allocating, concealing, and blinding. Moreover, variations were observed in the average age of participants, intervention duration, types of adjunctive pharmacotherapy utilized, and outcome assessment criteria across the studies included in this analysis. Furthermore, the reports of adverse events in the included literature are not comprehensive, and more rigorous studies are needed in the future to monitor the relevant outcome indicators to fully demonstrate the safety of the NSTC. Finally, despite conducting database searches without language restrictions, all included studies originated from China, thus lacking geographical diversity. These limitations collectively influence the reliability of the findings, thereby restricting the universality of the analytic results of the study.

## 5 Conclusion

This meta-analysis study revealed the difference in the efficacy of the NSTC in different periods of stroke and the value of secondary prevention, which helps further optimize the stroke treatment process and guide doctors to provide appropriate treatment within the best time window, thus expanding the scope of choice for doctors in clinical practice. At the same time, this study may promote the updating and improvement of relevant treatment guidelines and promote the standardization and standardization of stroke treatment. Given the low quality of the evidence so far, in the future, further large-scale, high-quality, and meticulously designed RCTs are warranted to validate these findings and make more efforts to confirm the contribution of the NSTC.

## Data Availability

The original contributions presented in the study are included in the article/Supplementary Material; further inquiries can be directed to the corresponding authors.

## Abbreviations

AE, adverse event; BI, Barthel Index; CNKI, China National Knowledge Internet; CI, confidence interval; GRADE, Grading of Recommendations, Assessment, Development, and Evaluation; IS, ischemic stroke; MD, mean difference; MBI, modified Barthel Index; mRS, modified Rankin Scale; NSTC, Naoshuantong capsule; NIHSS, National Institutes of Health Stroke Scale; PRISMA, Preferred Reporting Items for Systematic Reviews; RCTs, randomized controlled trials; RR, relative risk; SS-QOL, Stroke-specific Quality of life; VIP, Chinese VIP information.
