# Supplementary material for: Updated evidence of the Naoshuantong capsule against ischemic stroke: a systematic review and meta-analysis of randomized controlled trials
Source: Front Pharmacol. 2024 Sep 26;15:1434764. doi: 10.3389/fphar.2024.1434764 (PMC11464442; doi:10.3389/fphar.2024.1434764)
Supplement: Supplementary file 1 [file DataSheet1.docx]

Supplementary Material

#### **◆ Section I**

Supplementary Table 1. Standard formulation of Naoshuantong capsules

The preparation of Naoshuantong capsules

Supplementary Figure 1. High performance liquid chromatograph of Naoshuantong capsules

#### **◆ Section II**

Supplementary Table 2. Preferred Reporting Items for Systematic Reviews and Meta-Analyses (PRISMA) 2020 checklist

#### **◆ Section Ⅲ**

#### Supplementary Table 3. Searching strategies

#### **◆ Section Ⅳ**

Supplementary Table 4. Adverse reactions

#### **◆ Section Ⅴ**

Supplementary Figure S1. Risk of bias summary

Supplementary Figure S2. Forest plot of sensitivity analysis of acute period of NIHSS

Supplementary Figure S3. Forest plot of subgroup analysis of the intervention on acute period of NIHSS

Supplementary Figure S4. Forest plot of sensitivity analysis of non-acute period of NIHSS

Supplementary Figure S5. Forest plot of sensitivity analysis of mRS

Supplementary Figure S6. Forest plot of sensitivity analysis of BI

Supplementary Figure S7. Forest plot of subgroup analysis of the period on BI

Supplementary Figure S8. Forest plot of subgroup analysis of the intervention on BI

Supplementary Figure S9. Forest plot of subgroup analysis of the duration of treatment on BI

Supplementary Figure S10. Forest plot of subgroup analysis of the period on MBI

Supplementary Figure S11. Forest plot of subgroup analysis of the duration of treatment on MBI

Supplementary Figure S12. The Egger’s test of NIHSS (acute period)

Supplementary Figure S13. The Egger’s test of BI

Supplementary Figure S14. The Egger’s test of AE

**Section Ⅰ.**

**Supplementary Table 1. Standard formulation of Naoshuantong capsules**

| **Pinyin name** | **Complete species name** | **Authorities /family name** | **Scientific name** | **Proportion (g)** |
| --- | --- | --- | --- | --- |
| Pu Huang | *Typha angustifolia L.* | Typhaceae | Typhae Pollen | 0.890 |
| Chi Shao | *Paeonia lactiflora Pall.* | Paeoniaceae | Paeoniae Radix Rubra | 0.635 |
| Yu Jin | *Curcuma longa L.* | Zingiberaceae | Curcumae Radix | 0.510 |
| Tian Ma | *Gastrodia elata Blume* | Orchidaceae | Gastrodiae Rhizoma | 0.255 |
| Lou Lu | *Leuzea uniflora (L.) Holub* | Asteraceae | Rhapontici Radix | 0.380 |

**The preparation of Naoshuantong capsules**

NSTC (batch No.: 160510) were provided by Guangdong Huanan Pharmaceutical Co., Ltd. (Dongguan, China). The product (TP, 33.33%; PR, 23.78%; CR, 19.10%; GR, 9.55%; RR, 14.23%) was produced according to the 2020 Chinese Pharmacopoeia (CP) as follows. 1) Ethanol (70%) was added to PR which was heated and extracted twice for 1 h each time. Then the extracts were combined and filtered after ethanol was recovered from the filtrates, and the mixture was concentrated to an appropriate amount, dried, pulverized, and added with an appropriate amount of calcium hydrogen phosphate. They were mixed and dried for later use. CR was added with 80% ethanol and heated twice under reflux for 1 h each time. The mixed extracts were filtered for further use. The dregs of PR and CR, TP (confined in gauze bags), GR, and RR were boiled in water twice, 1 h each time, to obtain decoction which was then filtered, and the filtrate was concentrated to a clear paste with a relative density of 1.04 to 1.10 (40 °C). The ethanol was added to make the alcohol content reach 70%; Afterward, the supernatant was collected and mixed with the alcohol extract of CR. After ethanol was recovered, the mixture was concentrated to an appropriate amount, dried, crushed, and added with an appropriate amount of calcium hydrogen phosphate. After PR dry powders were added, it was granulated with hypromellose ethanol solution, dried, and mixed with talc, silicon dioxide and magnesium stearate to make capsules.

**Reference:**

Luo L, Wu S, Chen R, Rao H, Peng W, Su W. The study of neuroprotective effects and underlying mechanism of Naoshuantong capsule on ischemia stroke mice. Chin Med. 2020 Nov 17;15(1):119. doi: 10.1186/s13020-020-00399-7.

National Pharmacopoeia Commission. Pharmacopoeia of the People's Republic of China 2020 Edition. Beijing: China Medical Science and Technology Press, 2020:1945-1946.

**Supplementary Figure 1. High performance liquid chromatograph of Naoshuantong capsules**


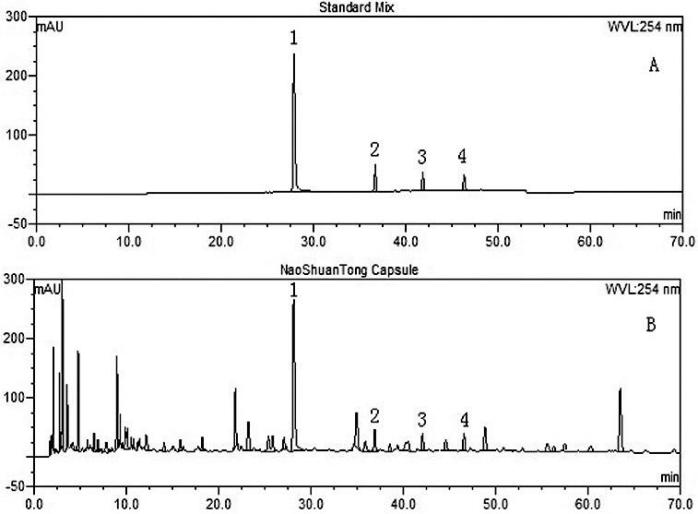


High performance liquid chromatograph of standard mix and naoshuantong capsules using ultraviolet absorbance detection at wavelength 254 nm.

1: Paeoniflorin; 2: Ecdysterone; 3: Typhaneoside; 4: Isorhamnetin-3-O-neohesperidoside.

**Reference:**

Liu H, Peng YY, Liang FY, et al. Protective effects of traditional Chinese medicine formula NaoShuanTong capsule on haemorheology and cerebral energy metabolism disorders in rats with blood stasis. Biotechnol Biotechnol Equip. 2014 Jan 2;28(1):140-146. doi: 10.1080/13102818.2014.901678

**
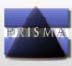
Section Ⅱ. PRISMA 2020 Checklist**

Supplementary Table 2. Preferred Reporting Items for Systematic Reviews and Meta-Analyses (PRISMA) 2020 checklist

| **Section and Topic** | **Item #** | **Checklist item** | **Location where item is reported** |
| --- | --- | --- | --- |
| **TITLE** | | |  |
| Title | 1 | Identify the report as a systematic review. |  |
| **ABSTRACT** | | |  |
| Abstract | 2 | See the PRISMA 2020 for Abstracts checklist. |  |
| **INTRODUCTION** | | |  |
| Rationale | 3 | Describe the rationale for the review in the context of existing knowledge. |  |
| Objectives | 4 | Provide an explicit statement of the objective(s) or question(s) the review addresses. |  |
| **METHODS** | | |  |
| Eligibility criteria | 5 | Specify the inclusion and exclusion criteria for the review and how studies were grouped for the syntheses. |  |
| Information sources | 6 | Specify all databases, registers, websites, organisations, reference lists and other sources searched or consulted to identify studies. Specify the date when each source was last searched or consulted. |  |
| Search strategy | 7 | Present the full search strategies for all databases, registers and websites, including any filters and limits used. |  |
| Selection process | 8 | Specify the methods used to decide whether a study met the inclusion criteria of the review, including how many reviewers screened each record and each report retrieved, whether they worked independently, and if applicable, details of automation tools used in the process. |  |
| Data collection process | 9 | Specify the methods used to collect data from reports, including how many reviewers collected data from each report, whether they worked independently, any processes for obtaining or confirming data from study investigators, and if applicable, details of automation tools used in the process. |  |
| Data items | 10a | List and define all outcomes for which data were sought. Specify whether all results that were compatible with each outcome domain in each study were sought (e.g. for all measures, time points, analyses), and if not, the methods used to decide which results to collect. |  |
|  | 10b | List and define all other variables for which data were sought (e.g. participant and intervention characteristics, funding sources). Describe any assumptions made about any missing or unclear information. |  |
| Study risk of bias assessment | 11 | Specify the methods used to assess risk of bias in the included studies, including details of the tool(s) used, how many reviewers assessed each study and whether they worked independently, and if applicable, details of automation tools used in the process. |  |
| Effect measures | 12 | Specify for each outcome the effect measure(s) (e.g. risk ratio, mean difference) used in the synthesis or presentation of results. |  |
| Synthesis methods | 13a | Describe the processes used to decide which studies were eligible for each synthesis (e.g. tabulating the study intervention characteristics and comparing against the planned groups for each synthesis (item #5)). |  |
|  | 13b | Describe any methods required to prepare the data for presentation or synthesis, such as handling of missing summary statistics, or data conversions. | NA |
|  | 13c | Describe any methods used to tabulate or visually display results of individual studies and syntheses. |  |
|  | 13d | Describe any methods used to synthesize results and provide a rationale for the choice(s). If meta-analysis was performed, describe the model(s), method(s) to identify the presence and extent of statistical heterogeneity, and software package(s) used. |  |
|  | 13e | Describe any methods used to explore possible causes of heterogeneity among study results (e.g. subgroup analysis, meta-regression). |  |
|  | 13f | Describe any sensitivity analyses conducted to assess robustness of the synthesized results. |  |
| Reporting bias assessment | 14 | Describe any methods used to assess risk of bias due to missing results in a synthesis (arising from reporting biases). |  |
| Certainty assessment | 15 | Describe any methods used to assess certainty (or confidence) in the body of evidence for an outcome. |  |
| **RESULTS** | | |  |
| Study selection | 16a | Describe the results of the search and selection process, from the number of records identified in the search to the number of studies included in the review, ideally using a flow diagram. |  |
|  | 16b | Cite studies that might appear to meet the inclusion criteria, but which were excluded, and explain why they were excluded. |  |
| Study characteristics | 17 | Cite each included study and present its characteristics. |  |
| Risk of bias in studies | 18 | Present assessments of risk of bias for each included study. |  |
| Results of individual studies | 19 | For all outcomes, present, for each study: (a) summary statistics for each group (where appropriate) and (b) an effect estimate and its precision (e.g. confidence/credible interval), ideally using structured tables or plots. |  |
| Results of syntheses | 20a | For each synthesis, briefly summarise the characteristics and risk of bias among contributing studies. |  |
|  | 20b | Present results of all statistical syntheses conducted. If meta-analysis was done, present for each the summary estimate and its precision (e.g. confidence/credible interval) and measures of statistical heterogeneity. If comparing groups, describe the direction of the effect. |  |
|  | 20c | Present results of all investigations of possible causes of heterogeneity among study results. |  |
|  | 20d | Present results of all sensitivity analyses conducted to assess the robustness of the synthesized results. |  |
| Reporting biases | 21 | Present assessments of risk of bias due to missing results (arising from reporting biases) for each synthesis assessed. |  |
| Certainty of evidence | 22 | Present assessments of certainty (or confidence) in the body of evidence for each outcome assessed. |  |
| **DISCUSSION** | | |  |
| Discussion | 23a | Provide a general interpretation of the results in the context of other evidence. |  |
|  | 23b | Discuss any limitations of the evidence included in the review. |  |
|  | 23c | Discuss any limitations of the review processes used. |  |
|  | 23d | Discuss implications of the results for practice, policy, and future research. |  |
| **OTHER INFORMATION** | | |  |
| Registration and protocol | 24a | Provide registration information for the review, including register name and registration number, or state that the review was not registered. |  |
|  | 24b | Indicate where the review protocol can be accessed, or state that a protocol was not prepared. | NA |
|  | 24c | Describe and explain any amendments to information provided at registration or in the protocol. | NA |
| Support | 25 | Describe sources of financial or non-financial support for the review, and the role of the funders or sponsors in the review. |  |
| Competing interests | 26 | Declare any competing interests of review authors. |  |
| Availability of data, code and other materials | 27 | Report which of the following are publicly available and where they can be found: template data collection forms; data extracted from included studies; data used for all analyses; analytic code; any other materials used in the review. | NA |

**Section Ⅲ. Searching strategies**

**Supplementary Table 3. For retrieval of databases, the keywords of search are the same, with taking pubmed as an example:**

| **No** | **Search items** | **No** | **Search items** |
| --- | --- | --- | --- |
| 1 | ischemic stroke | 16 | controlled clinical trial |
| 2 | Ischemic Strokes | 17 | randomized |
| 3 | Stroke, Ischemic | 18 | Placebo |
| 4 | Ischaemic Stroke | 19 | clinical trials as topic |
| 5 | Ischaemic Strokes | 20 | randomly |
| 6 | Cerebral Infarction | 21 | trial |
| 7 | Brain Infarction | **22** | **15-21** |
| 8 | cerebral embolism | **23** | **10 AND 14 AND 22** |
| 9 | brain embolism |  |  |
| **10** | **1 OR 2-9** |  |  |
| 11 | Naoshuantong |  |  |
| 12 | Nao-shuan-tong |  |  |
| 13 | Nao shuan tong |  |  |
| **14** | **11 OR 12-13** |  |  |
| 15 | randomized controlled trial |  |  |

**Section Ⅳ.**

**Supplementary Table 4. Adverse reactions**

| Study | Intervention | Physical therapy | Experimental group | Control group |
| --- | --- | --- | --- | --- |
| Chen et al, 2021 | NSTC 1.2g Tid po + Aspirin + Atorvastatin | Aspirin + Atorvastatin | - | 1 patient had nausea and 1 patient had vomiting |
| Fu et al,2021 | NSTC 1.2g Tid po + Butylphthalide | Butylphthalide | Rash occurred in 3 patients, somnolence in 2 patients, nausea in 3 patients, and the abnormal level of transaminase in 1 patient | Rash in 1 patient, somnolence in 1 patient, and nausea in 1 patient |
| Huang et al,2020 | NSTC 1.2g Tid po + Yurekline | Yurekline | - | - |
| Li et al,2020 | NSTC 1.2g Tid po + Yurekline | Yurekline | Nausea and vomiting were found in 1 patient, abdominal pain and diarrhea were found in 1 patient | 2 patients had nausea and vomiting, 1 patient had abdominal pain and diarrhea, and 1 patient had serum creatinine increasing |
| Lu et al,2020 | NSTC 1.2g Tid po + Butylphthalide | Butylphthalide | 1 patient had nausea and 1 patient had vomiting | 1 patient had nausea and 1 patient had vomiting |
| Luo 2022 | NSTC 1.2g Tid po + Clopidogrel | Clopidogrel | Gastrointestinal reaction occurred in 2 patients, allergic reaction in 1 patient, chest tightness and palpitation in 1 patient | Gastrointestinal reaction occurred in 4 patients, allergic reaction in 3 patients, chest tightness and palpitation in 3 patients, and arrhythmia in 2 patients |
| Wang et al,2015 | NSTC 1.2g Tid po + Clopidogrel | Clopidogrel | - | 1 patient had the fecal occult blood test positive, the other 1 patient had nausea and vomiting with abdominal distension |
| Wang et al,2021 | NSTC 1.2g Tid po + Aspirin + Clopidogrel + rt-PA | Aspirin + Clopidogrel + rt-PA | Nausea and vomiting occurred in 2 patients, rash in 1 patient, and dizziness in 1 patient | Nausea and vomiting occurred in 3 patients, rash in 2 patients, dizziness in 2 patients, and liver and kidney injury in 1 patient |
| Wu 2021 | NSTC 1.2g Tid po + Butylphthalide | Butylphthalide | Abdominal pain and diarrhea were found in 1 patient, and the high level of transaminase was found in 1 patient | Nausea and vomiting occurred in 1patient, abdominal pain and diarrhea in 1patient, and the high level of transaminase was found in 2 patients |
| Ye et al,2  015 | NSTC 1.2g Tid po + Aspirin | Aspirin | 1 patient died from a car accident， symptomatic epilepsy happened in 1 patient, and 12 patients underwent the abnormal liver function | Acute heart failure in 1 patient, cerebral hemorrhage in 1 patient, pulmonary infection in 1 patient, and 8 patients underwent the abnormal liver function |

**Section Ⅴ.**

**Supplementary Figure S1. Risk of bias graph**

**
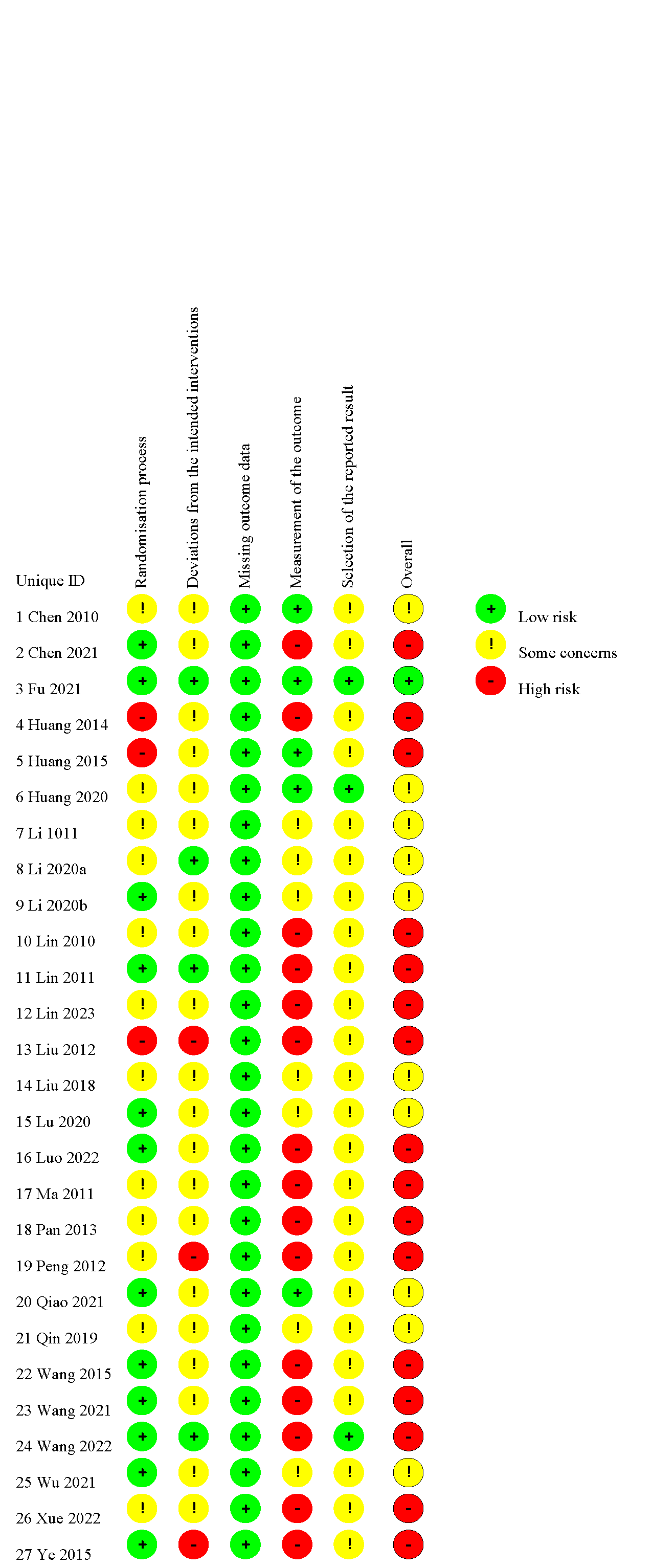
**

**Supplementary Figure S2. Forest plot of sensitivity analysis of acute period of NIHSS**

**
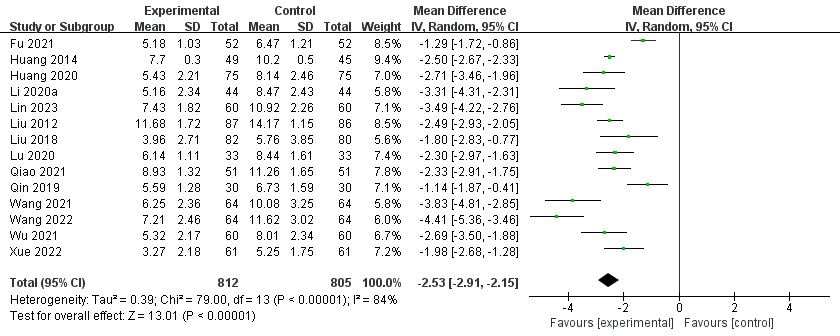
**

**Supplementary Figure S3. Forest plot of subgroup analysis of the intervention on acute period of NIHSS**

**
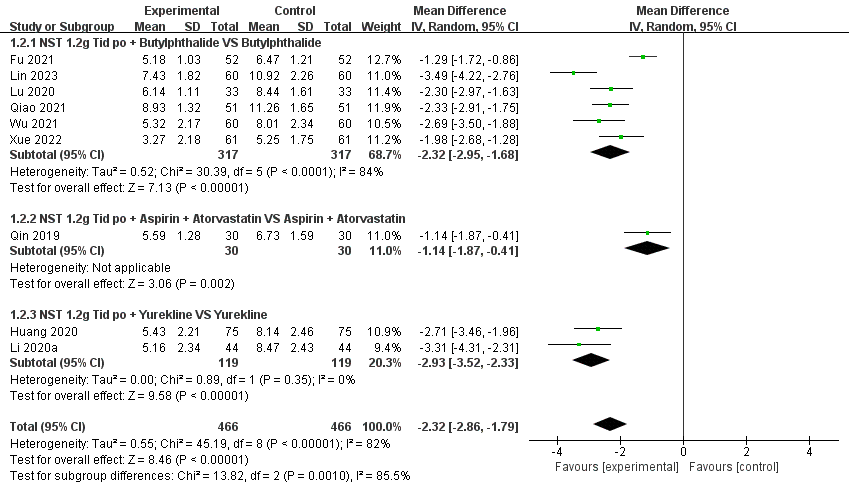
**

**Supplementary Figure S4. Forest plot of sensitivity analysis of non-acute period of NIHSS**

**
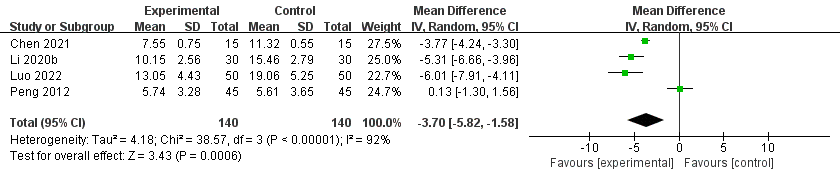
**

**Supplementary Figure S5. Forest plot of sensitivity analysis of mRS**

**
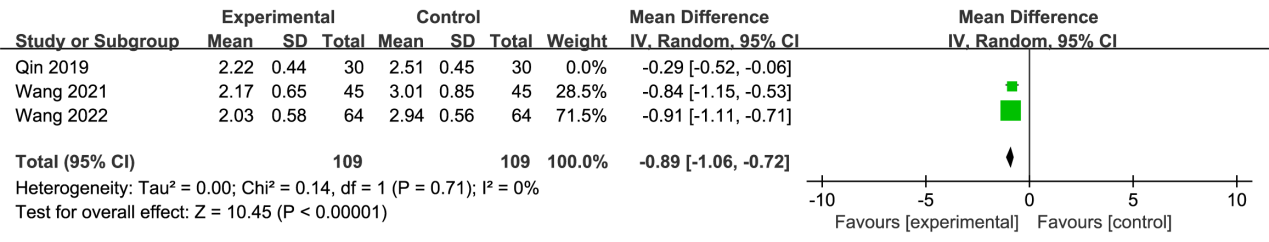
**

**Supplementary Figure S6. Forest plot of sensitivity analysis of BI**

**
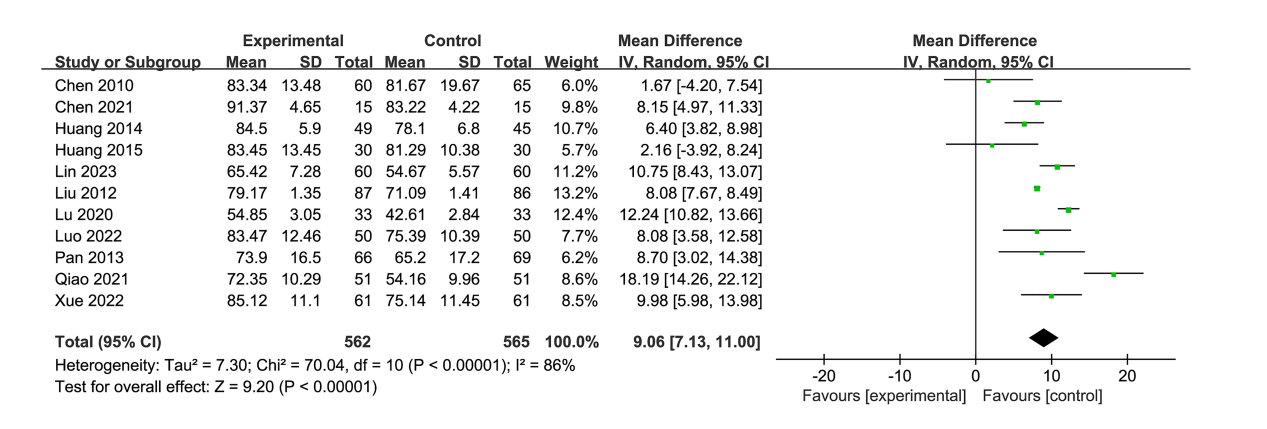
**

**Supplementary Figure S7. Forest plot of subgroup analysis of the period on BI**

**
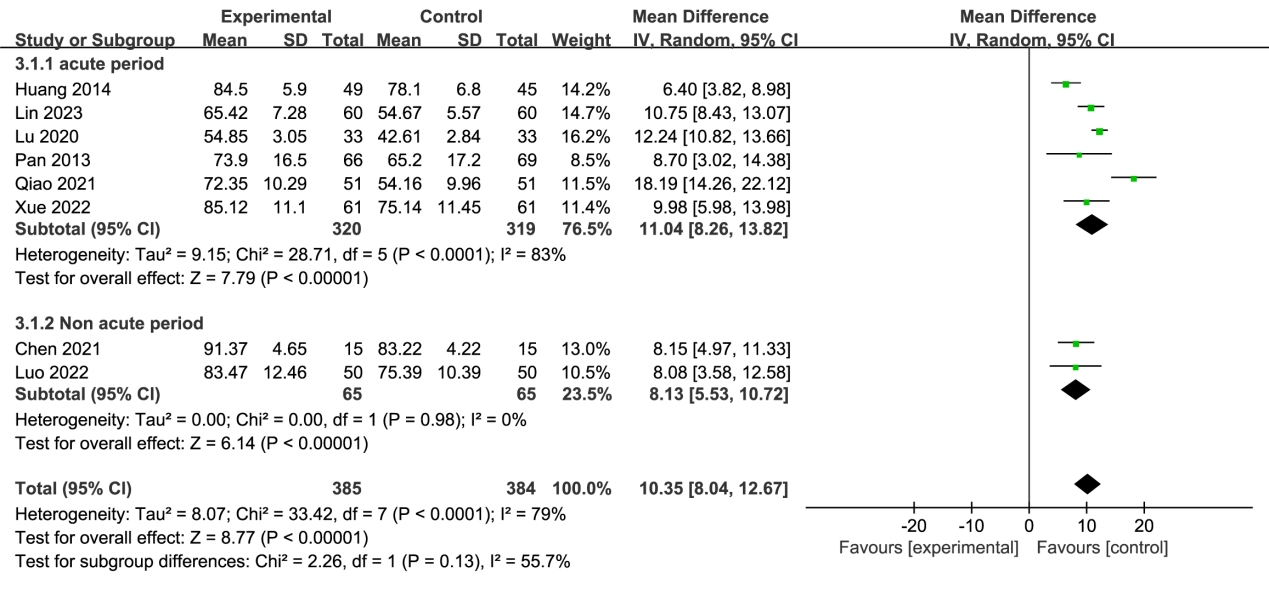
**

**Supplementary Figure S8. Forest plot of subgroup analysis of the intervention on BI**

**
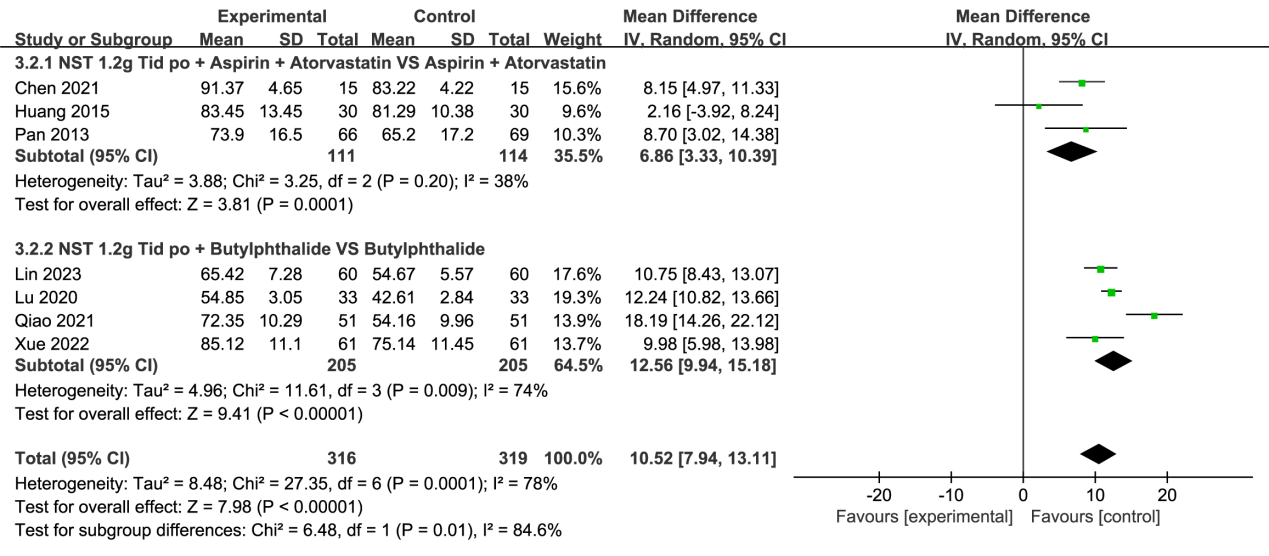
**

**Supplementary Figure S9. Forest plot of subgroup analysis of the duration of treatment on BI**

**
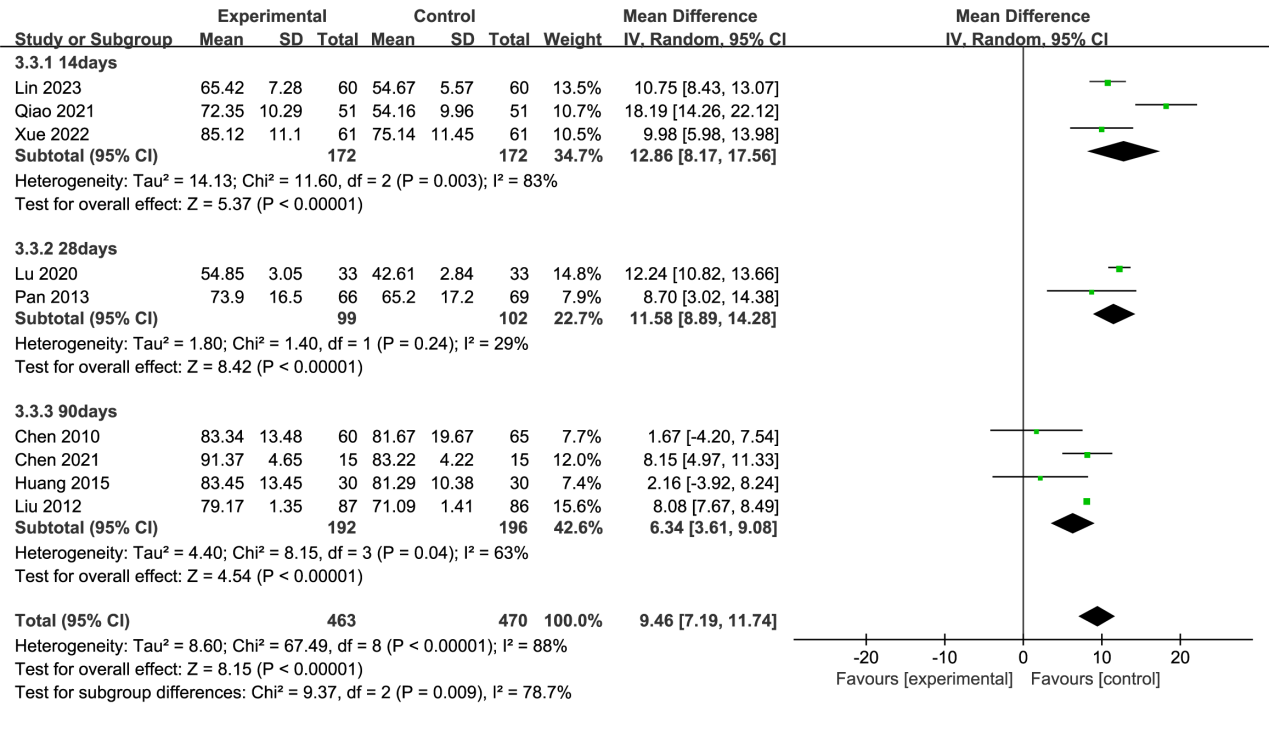
**

**Supplementary Figure S10. Forest plot of subgroup analysis of the period on MBI**

**
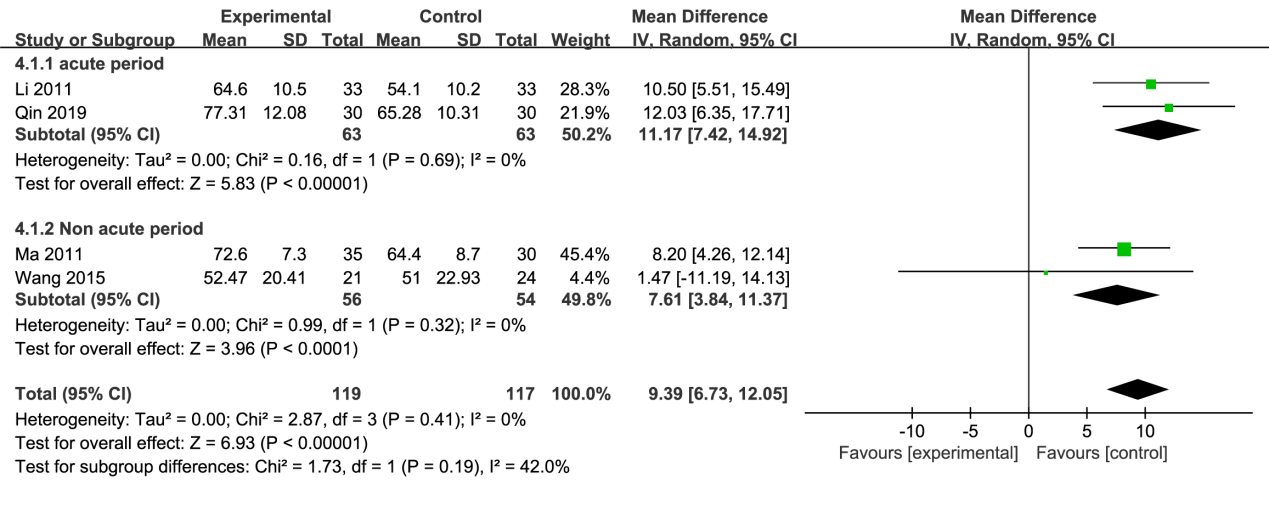
**

**Supplementary Figure S11. Forest plot of subgroup analysis of the duration of treatment on MBI**

**
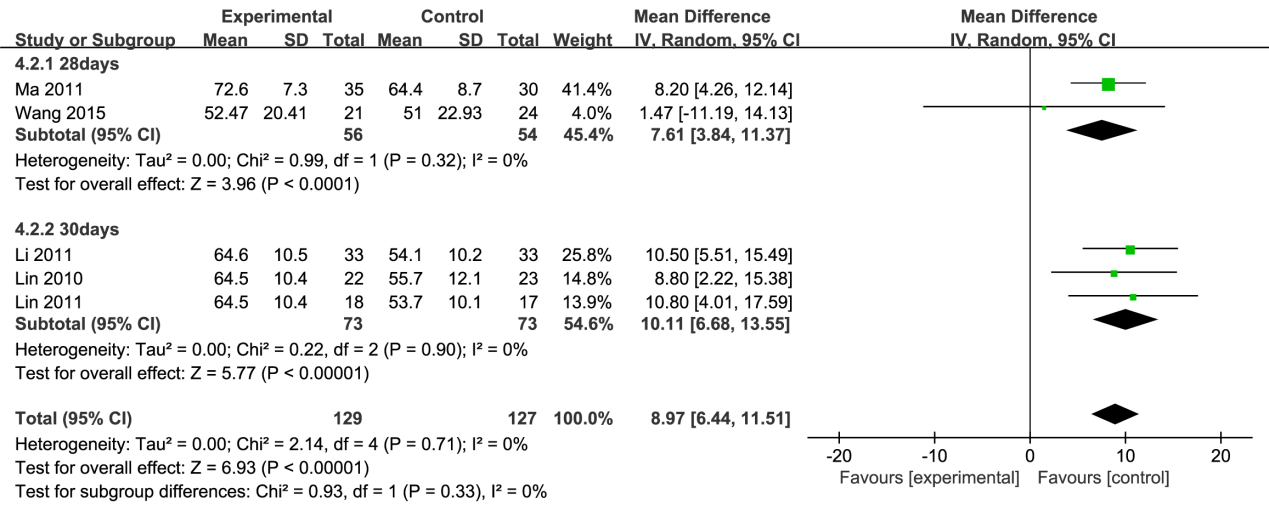
**

**Supplementary Figure S12. The Egger’s test of NIHSS****(acute period)**

**
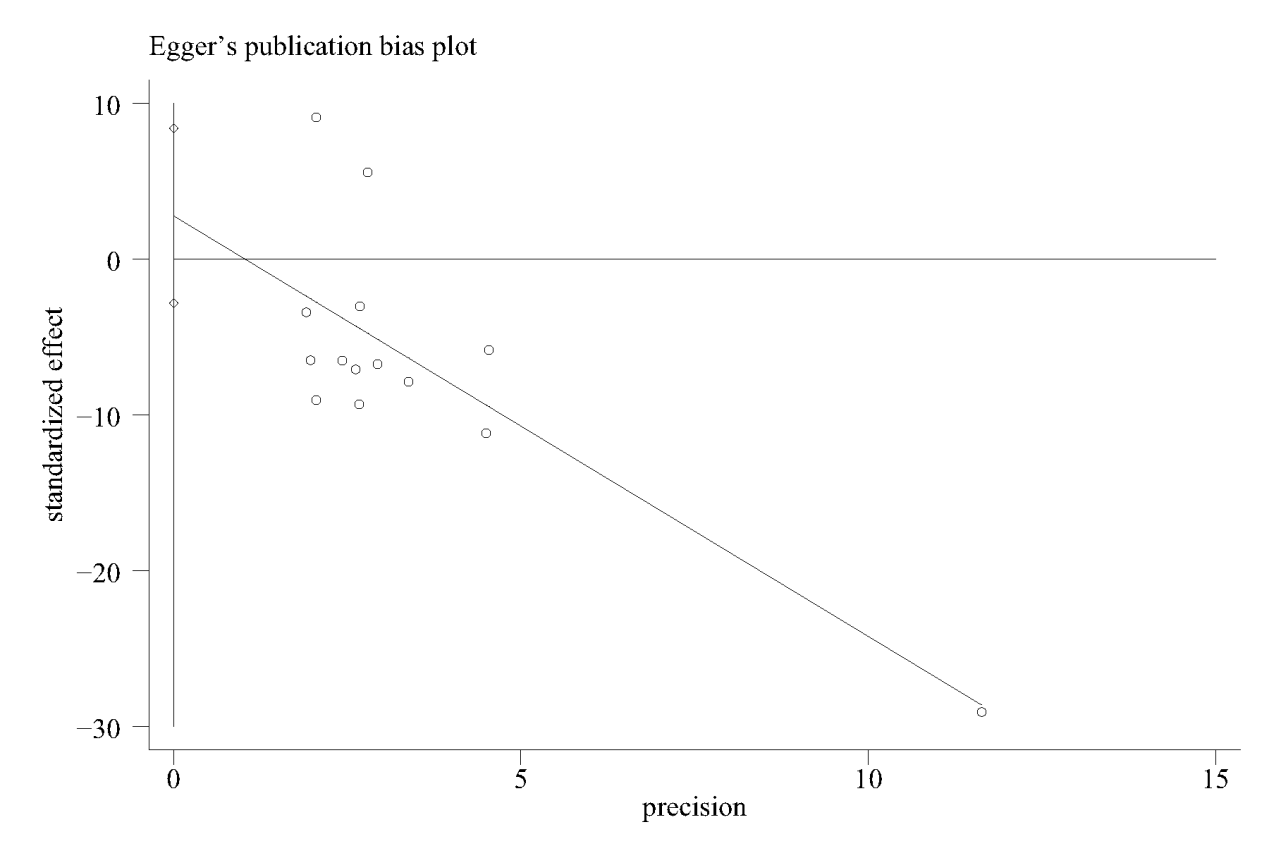
**

**Supplementary Figure S13. The Egger’s test of BI**

**
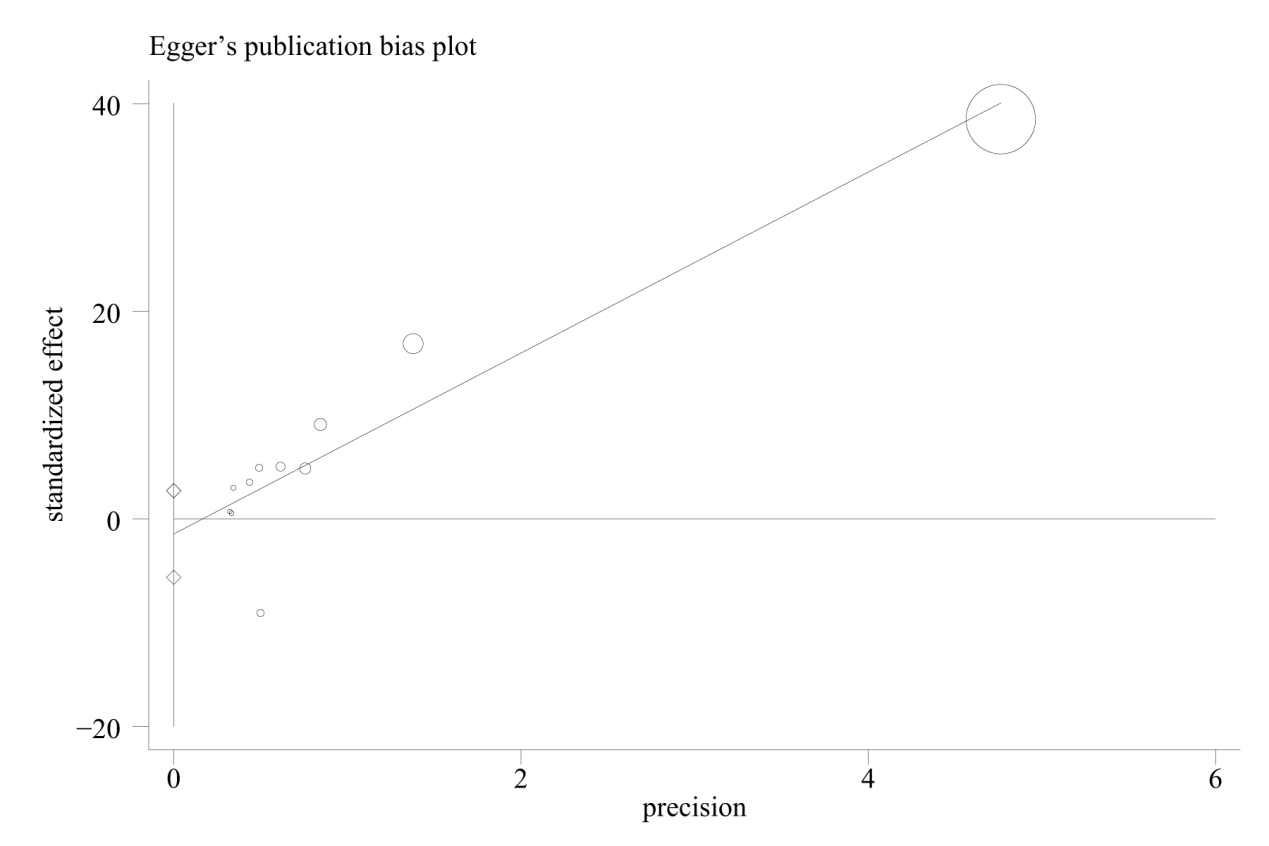
**

**Supplementary Figure S14. The Egger’s test of AE**

**
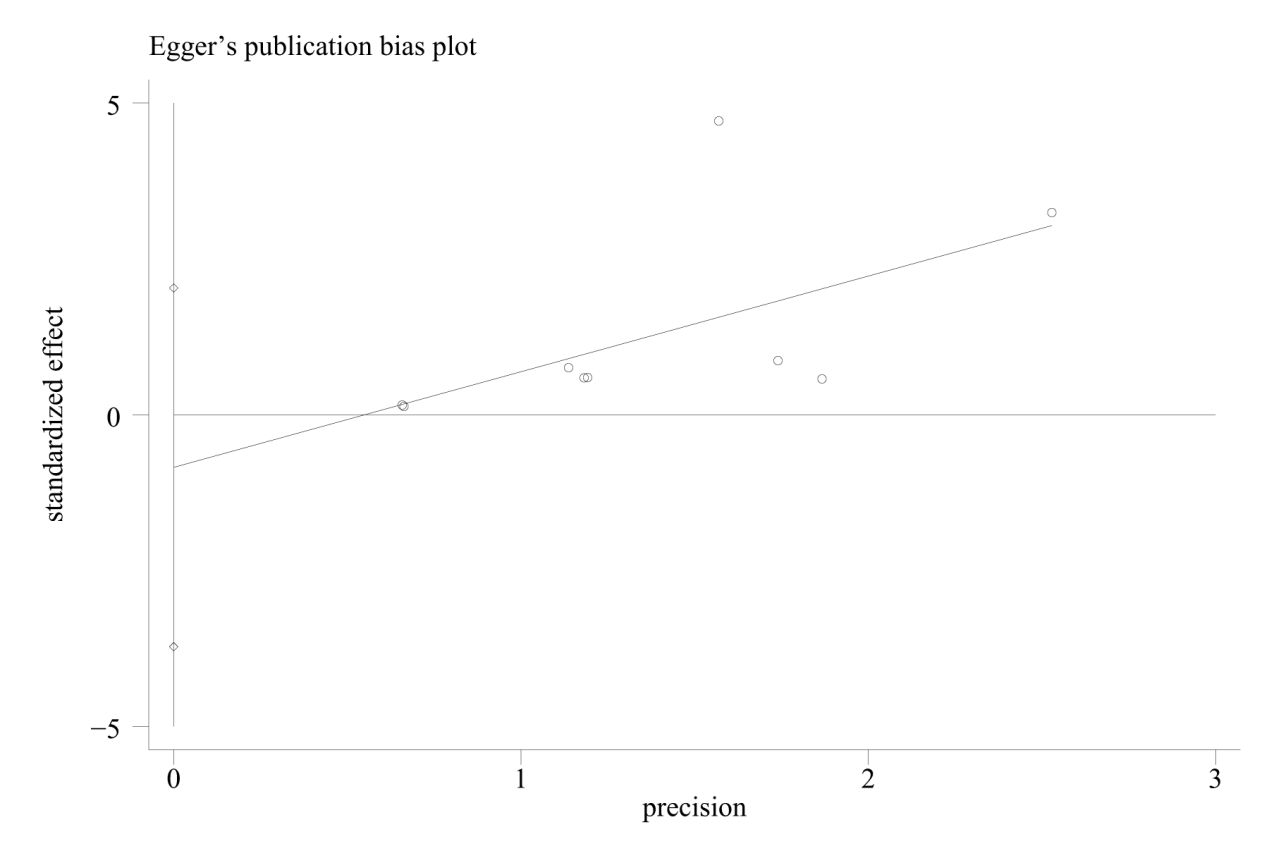
**
